# TERRA transcripts localize at long telomeres to regulate telomerase access to chromosome ends

**DOI:** 10.1126/sciadv.adk4387

**Published:** 2024-06-12

**Authors:** Nicole Bettin, Emmanuelle Querido, Irene Gialdini, Glenda Paola Grupelli, Elena Goretti, Marta Cantarelli, Marta Andolfato, Eslam Soror, Alessandra Sontacchi, Katarina Jurikova, Pascal Chartrand, Emilio Cusanelli

**Affiliations:** ^1^ Laboratory of Cell Biology and Molecular Genetics, Department CIBIO, University of Trento, via Sommarive 9, 38123 Trento, Italy.; ^2^ Department of Biochemistry and Molecular Medicine, University of Montreal, 2900 boul. Edouard Montpetit, H3T1J4 Montreal, Canada.; ^3^ Department of Genetics, Faculty of Natural Sciences, Comenius University in Bratislava, Ilkovičova 6, Mlynská dolina, 84215 Bratislava, Slovakia.

## Abstract

The function of TERRA in the regulation of telomerase in human cells is still debated. While TERRA interacts with telomerase, how it regulates telomerase function remains unknown. Here, we show that TERRA colocalizes with the telomerase RNA subunit hTR in the nucleoplasm and at telomeres during different phases of the cell cycle. We report that TERRA transcripts relocate away from chromosome ends during telomere lengthening, leading to a reduced number of telomeric TERRA-hTR molecules and consequent increase in “TERRA-free” telomerase molecules at telomeres. Using live-cell imaging and super-resolution microscopy, we show that upon transcription, TERRA relocates from its telomere of origin to long chromosome ends. Furthermore, TERRA depletion by antisense oligonucleotides promoted hTR localization to telomeres, leading to increased residence time and extended half-life of hTR molecules at telomeres. Overall, our findings indicate that telomeric TERRA transcripts inhibit telomere elongation by telomerase acting in trans, impairing telomerase access to telomeres that are different from their chromosome end of origin.

## Teaser

TERRA transcripts inhibit telomere elongation by impairing telomerase access to chromosome ends.

## INTRODUCTION

Long noncoding telomeric repeat-containing RNA (TERRA) is transcribed from telomeres in several organisms including humans, yeasts, and nematodes (*1*–*4*). Transcription starts within subtelomeric sequences and proceeds toward the extremity of the chromosomes, terminating within their telomeric repeat tract (*5*). As a result, TERRA molecules consist of subtelomeric-derived sequences at their 5′ end and G-rich telomeric repeats at their 3′ extremity (*6*).

In human cells, TERRA promoters lay in close proximity or at 5 to 10 kb from the telomeric repeats of chromosome ends (*7*, *8*). TERRA transcription is regulated by the chromatin state of telomeres and subtelomeric regions in a telomere-specific manner (*9*).

TERRA transcripts localize to chromosome ends, and their localization is controlled by multiple mechanisms. Components of the nonsense-mediated mRNA decay (NMD) pathway (*1*) and members of the heterogeneous nuclear ribonucleoprotein family (hnRNPs) (*10*) can actively displace TERRA from telomeres, while expression of a mutant form of the telomeric repeat-binding factor 2 (TRF2), a subunit of the shelterin protein complex that is assembled at telomeres, results in TERRA delocalization from chromosome ends (*11*). Despite this evidence, little is known on the dynamics of TERRA molecules in human cells, where it remains to be defined whether the endogenous transcripts preferentially localize in cis, to their telomere of origin, as previously observed in yeast (*12*, *13*), or in trans, by relocating to other telomeres. The telomeric repeat tract of the transcripts is important for their recruitment to telomeres as it can coordinate the interaction with telomere-binding proteins as well as formation of telomeric R-loop structures (*14*). The presence of the poly(A) tail, which is detected in about 10% of TERRA molecules in human cells (*15*), influences TERRA distribution within the nucleus. Nonpolyadenylated TERRA is found within the nucleoplasm and associated with chromatin, while poly(A)^+^ TERRA molecules are predominantly nucleoplasmic (*16*). While the mechanism of TERRA polyadenylation remains to be elucidated, recent evidence indicates that TERRA transcripts are polyadenylated in a telomere-specific manner (*17*).

The poly(A) tail, as well as the 5′ sequence of TERRA, is also important for its stability. TERRA molecules are m^6^A-modified within their subtelomeric and telomeric sequences, a modification that stabilizes the transcripts (*18*, *19*). Furthermore, the TERRA-binding proteins RBP associated with lethal yellow mutation (RALY) and poly(A)-binding protein nuclear 1 (PABPN1) regulate the stability of TERRA through a modality that is dependent on the presence of the poly(A) tail. Nonpolyadenylated TERRA transcripts are stabilized by RALY, while they can be degraded through a mechanism that is mediated by PABPN1 (*17*).

At chromosome ends, TERRA has been proposed to model chromatin (*20*), promote proper capping of telomeres (*21*), and act as a scaffold to assist the recruitment of TERRA-binding proteins, thereby participating in DNA replication (*11*, *22*) and activation of DNA damage response pathways (*8*, *23*). Furthermore, an important function of TERRA is emerging in telomere length homeostasis. Progressive shortening of chromosome ends during subsequent cell divisions is inevitable due to the inability of the DNA replication machinery to fully replicate the extremities of eukaryotic chromosomes, and as a result of exonucleolytic activities (*24*). Critically short telomeres trigger an irreversible cell cycle arrest, known as replicative senescence (*25*). In most eukaryotes, telomere erosion is counteracted by the activity of telomerase, a reverse transcriptase enzyme that uses its RNA subunit as a template for the addition of telomeric DNA to the 3′ end of chromosomes (*26*, *27*). Telomere elongation by telomerase takes place during the S phase of the cell cycle (*27*). Telomerase is not active in human somatic cells, which enter senescence upon a defined number of cell divisions, while it is re-activated in about 90% of human tumors, enabling cancer cell immortality (*28*).

In human cells, the recruitment of telomerase to chromosome ends is mediated by the direct interaction between TNT1/PTOP/PIP1 (TPP1), a component of shelterin, and human telomerase reverse transcriptase (hTERT), the catalytic subunit of telomerase (*29*, *30*). The telomerase-telomere association is stabilized by the base pairing between the template region of human telomerase RNA (hTR), the RNA subunit of telomerase, and the G-rich single-stranded DNA overhang that is present at the 3′ end of chromosomes (*31*). The shelterin component protection of telomeres (POT1) interacts with TPP1 and binds the single-stranded telomeric sequence (*32*, *33*). By this mechanism, POT1 can inhibit telomere elongation, preventing hTR binding to the telomeric overhang (*34*). However, the TPP1-POT1 complex has also been shown to stimulate telomerase processivity (*35*, *36*). The molecular details of telomerase regulation at telomeres are not fully elucidated. Recently, a live-cell imaging study of hTR unveiled a recruitment-retention model of telomere elongation by telomerase (*37*). Here, it was shown that expression of a mutant form of POT1 harboring deletion of its OB-fold DNA binding domain (POT1-ΔOB), preventing its interaction with the single-stranded telomeric DNA, enhances telomerase retention to chromosome ends (*37*). While in steady-state conditions most telomeres can be elongated by telomerase during a single cell division in human cancer cells (*38*), live imaging studies have shown that both hTERT and hTR form stable interactions only with a few telomeres at any given time of the S phase (*37*, *39*). The mechanisms that regulate telomerase distribution at telomeres remain to be defined.

TERRA interacts with telomerase and acts as a positive regulator of telomerase in yeasts (*13*, *40*). In *Saccharomyces cerevisiae*, TERRA transcripts can promote the clustering of telomerase molecules and colocalize with the telomerase RNA subunit at short telomeres to promote their elongation (*13*). To date, no studies have investigated TERRA-telomerase colocalizations in human cells. TERRA interacts with hTERT and hTR (*41*), yet the mechanisms of TERRA-mediated telomerase regulation in human cells remain to be determined. TERRA-mimicking RNA oligonucleotides inhibit telomerase activity in vitro (*2*, *41*, *42*), and TERRA down-regulation by antisense oligonucleotides (ASO) results in an increase of telomerase activity detected from total cell extracts of mouse embryonic stem cells (mESCs) (*43*). As the 3′ end of TERRA consists of G-rich telomeric sequences that are complementary to the template region of telomerase RNA, TERRA molecules may act as direct inhibitors of telomerase, preventing its base pairing to the telomeric overhang (*41*). However, despite these findings, the inducible expression of TERRA from a single telomere does not affect telomerase activity at this engineered chromosome end (*44*). Moreover, TERRA levels increase during generation of induced pluripotent stem cells (iPSCs), a process that requires telomerase activation and telomere elongation (*45*–*49*). The study of the role of TERRA in the regulation of telomerase is complicated by the dynamics of the enzyme that transiently localizes to telomeres during S phase (*50*, *51*).

To gain insight into the role of TERRA in telomerase regulation, we studied TERRA and telomerase localization during telomere length homeostasis. We show that during telomere lengthening, TERRA molecules relocate from telomeres to the nucleoplasm. Furthermore, using live-cell imaging approaches and super-resolution microscopy, we report that upon transcription, endogenous TERRA molecules move from their telomere of origin to localize to longer chromosome ends. By directly visualizing both TERRA transcripts and the telomerase RNA hTR through single-molecule inexpensive RNA fluorescence in situ hybridization (smiFISH), we found that a fraction of TERRA colocalizes with hTR both in the nucleoplasm and at telomeres. As opposed to the findings observed in yeast, we report that in human cells, the telomeric fraction of TERRA-hTR molecules markedly decreases during telomere elongation, while the number of telomeres with “TERRA-free” telomerase molecules rises. Accordingly, enhanced retention of telomerase at telomeres by expression of the mutant POT1-ΔOB protein leads to less TERRA transcripts at chromosome ends and increased number of TERRA-free telomerase molecules at telomeres, a condition associated with over-elongated telomeres. Finally, we report that down-regulation of TERRA by ASOs results in increased hTR clustering, enhanced localization of hTR at telomeres, and extended residence time and half-life of hTR molecules at chromosome ends. Together, our data support a model in which TERRA molecules coordinate telomerase access to chromosome ends.

## RESULTS

### TERRA and hTR colocalize at telomeres in human cancer cells

**Fig. 1.**
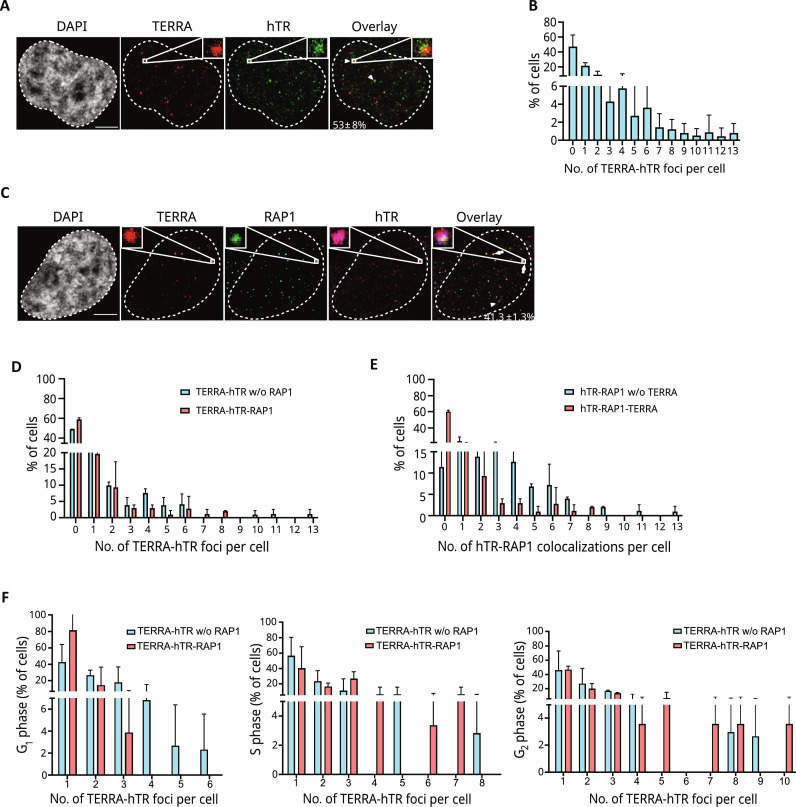
TERRA and hTR colocalize at telomeres in HeLa cells. (**A**) Detection of TERRA and hTR by smiFISH in HeLa cells. The percentage of cells, in which at least 1 TERRA-hTR colocalization event is detected, is indicated (mean ± SD; *n* = 5; 298 cells analyzed). Scale bar, 5 μm. (**B**) Quantification of the number of TERRA-hTR colocalizations per nucleus (mean ± SD; *n* = 5; 298 cells analyzed). (**C**) Detection of TERRA, hTR, and telomeres by smiFISH/RAP1 IF in HeLa cells (mean ± SD; *n* = 2; 102 cells analyzed). Scale bar, 5 μm. (**D**) Quantification of the number of TERRA-hTR colocalizations per cell detected at telomeres (TERRA-hTR-RAP1) and outside telomeres (TERRA-hTR w/o RAP1) (mean ± SD; *n* = 2; 102 cells analyzed). (**E**) Number of hTR-RAP1 colocalizations per cell with and w/o TERRA (mean ± SD; *n* = 2; 102 cells analyzed). (**F**) Quantification of the number of TERRA-hTR foci at telomeres (TERRA-hTR-RAP1) and outside telomeres (TERRA-hTR w/o RAP1) during G_1_, S, and G_2_ phase in HeLa cells upon cell synchronization (mean ± SD; *n* = 2; total number of cells analyzed: 54 (G_1_ phase), 46 (S phase), and 45 (G_2_ phase). Fraction of hTR-TERRA foci at telomeres: 20.9 ± 3.3% in G_1_-phase cells, 40.2 ± 11% in S-phase cells, and 41.1 ± 5.5% in G_2_-phase cells.

We used smiFISH to visualize TERRA and hTR molecules at single-cell resolution (*17*, *52*, *53*). As expected, both RNAs were detected as discrete foci predominantly nuclear and sensitive to ribonuclease (RNase) treatment (fig. S1A). TERRA and hTR colocalizing foci (hereafter also TERRA-hTR foci) were detected in approximately 53% of HeLa cells (Fig. 1A). Mostly one to four TERRA-hTR colocalizations were detected per cell, with approximately 20% of cells displaying more than five TERRA-hTR foci detected in a single cell (Fig. 1B). Similar results were observed in other cancer cell lines, suggesting conserved regulatory mechanisms (fig. S1B).

To study whether TERRA and hTR colocalize at telomeres, we performed smiFISH combined with immunofluorescence (IF) (smiFISH/IF) using an antibody specific for the telomere-binding protein and shelterin component repressor activator protein 1 (RAP1) (*54*, *55*). These experiments revealed that a fraction of TERRA-hTR foci resides at chromosome ends in more than 40% of cells (Fig. 1C). Most TERRA-hTR–positive cells displayed one or two telomeric colocalizations (Fig. 1D). Similarly, in these cells, mostly one to three telomeric hTR foci not colocalizing with TERRA were detected per nucleus (Fig. 1E). To gain information on the cell cycle phase at which TERRA-hTR foci colocalize at telomeres, we performed smiFISH/IF experiments in HeLa cells upon cell synchronization using double thymidine block. A similar number of TERRA-hTR foci were detected in G_1_-, S-, and G_2_-phase cells, while a higher number of telomeric TERRA-hTR foci per cell were observed during S and G_2_ phases of the cell cycle (Fig. 1F). These findings indicate that TERRA and hTR transcripts colocalize at telomeres. Telomeric TERRA-hTR foci were detected also during S phase, suggesting that TERRA molecules may participate in telomerase regulation.

### Telomeric localization of TERRA and TERRA-hTR foci inversely correlates with telomerase-dependent telomere elongation

**Fig. 2.**
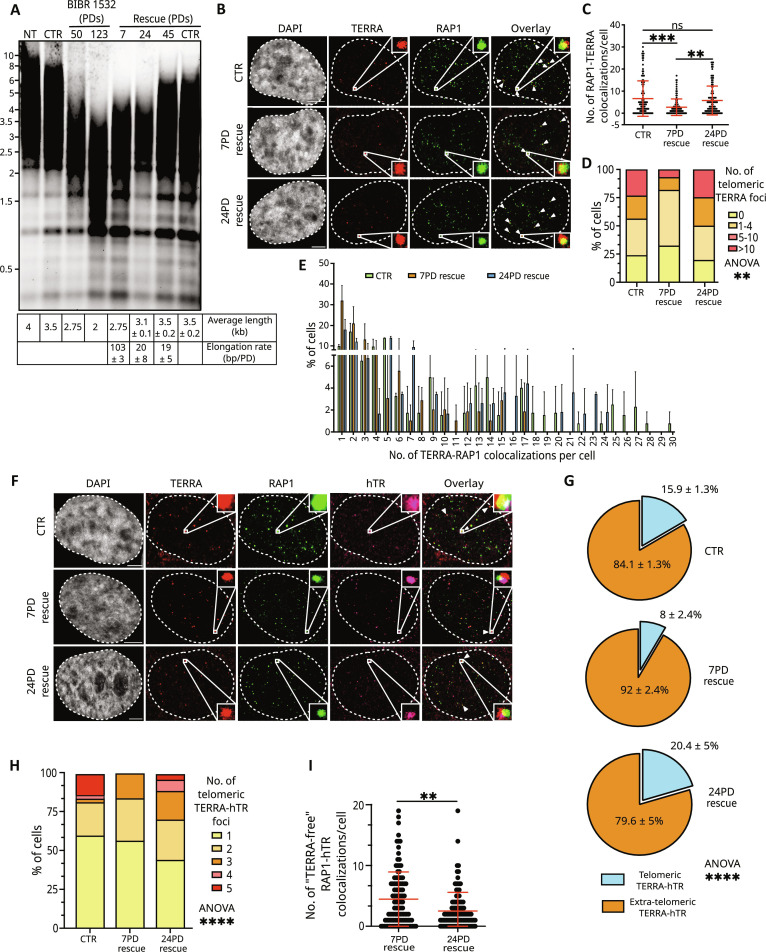
Telomeric localization of TERRA and TERRA-hTR foci inversely correlates with telomere elongation by telomerase. (**A**) Telomere length measurement by TRF through Southern blot in HeLa cells in the indicated conditions. NT, untreated; CTR, DMSO-treated. Estimated average telomere length and elongation rate for each sample are indicated in the table below. Average ± SD from two technical replicates. (**B**) Detection of TERRA and telomeres by smiFISH/IF in CTR, 7PD rescue, and 24PD rescue cells. Scale bar, 5 μm. (**C**) Quantification of the number of telomeric TERRA foci detected per nucleus by smiFISH/IF (each dot represents a nucleus) (mean ± SD; *n* = 2; 124 CTR cells, 111 7PD rescue cells, and 112 24PD rescue cells analyzed). Unpaired nonparametric Kruskal-Wallis test coupled with post hoc Dunn’s multiple-comparison test: **P < 0.01, ***P < 0.001. (**D** and **E**) Distribution analyses of the number of telomeric TERRA foci per cell. Two-way analysis of variance (ANOVA) test: **P < 0.01. Post hoc Tukey’s multiple-comparison test: group 1 to 4 *P* values = 0.07 (CTR versus 7PD rescue), 0.04 (7PD rescue versus 24PD rescue), not significant (ns) (CTR versus 24PD rescue). (**F**) Detection of TERRA, hTR, and telomeres by smiFISH/IF in CTR, 7PD rescue, and 24PD rescue cells. Scale bar, 5 μm. (**G**) Quantification of the percentage of TERRA-hTR foci colocalizing at telomeres and extratelomeric (mean ± SD; *n* = 2; 124 CTR cells, 111 7PDs rescue cells, and 112 24PDs rescue cells analyzed). Two-way ANOVA test: ****P < 0.0001. (**H**) Distribution analyses of the number of TERRA-hTR-RAP1 colocalizing foci per nucleus. Two-way ANOVA test: ****P < 0.0001; multiple-comparison test: *P* = 0.0003 for 7PD versus 24PD rescue, *P* < 0.0001 for CTR versus 7PD rescue, *P* = 0.001 CTR versus 24PD rescue. (**I**) Number of telomeric hTR foci not colocalizing with TERRA per cell detected by smiFISH/IF. Kruskal-Wallis test: *P* = 0.0025.

To gain insights into the function of TERRA in telomerase regulation, we investigated the telomeric localization of TERRA transcripts during telomere elongation. To this aim, we treated HeLa cells with BIBR1532, a well-known inhibitor of telomerase, to promote telomere shortening (*56*). Telomere restriction fragment (TRF) analyses by Southern blot confirmed a progressive decrease in telomere length during treatment for up to 123 population doublings (PDs), corresponding to 5 months in culture (Fig. 2A). Subsequent removal of BIBR1532 from the culturing medium enabled telomere re-elongation by the endogenous telomerase (rescue time points) (Fig. 2A), as previously reported in literature (*56*). Quantification of telomere size showed a telomere elongation rate of approximately 103 base pairs (bp)/PD at 7PD rescue, 20 bp/PD at 24PD rescue, and 19 bp/PD at 45PD rescue (Fig. 2A), indicating that telomere elongation is most prominent at the 7PD rescue time point, when the overall telomere length is shorter than the subsequent time points.

smiFISH/IF experiments revealed a decreased number of telomeric TERRA foci at 7PD rescue time point, compared to HeLa cells treated with dimethyl sulfoxide (DMSO) (CTR), and at 24PD rescue time point (Fig. 2, B and C). Accordingly, the percentage of cells displaying more than 10 telomeric TERRA foci was lower at the 7PD rescue time point, compared to CTR and 24PD rescue cells (Fig. 2, D and E). The total number of TERRA foci per cell remained unchanged between samples (fig. S2A). The total number of telomeres detected by RAP1 IF also did not differ between 7PD rescue and 24PD rescue time points (fig. S2B), while CTR cells displayed a higher number of telomeric foci, consistent with the presence of longer telomeres in these cells (fig. S2B). Northern blot and reverse transcription quantitative polymerase chain reaction (RT-qPCR) analyses revealed similar levels of TERRA between samples (fig. S2, C and D). These findings suggest that during telomere re-lengthening, the decreased detection of TERRA at telomeres is due to a delocalization of the transcripts from chromosome ends rather than their reduced expression. Notably, when we analyzed the fraction of TERRA-hTR foci at telomeres, we observed a decrease from ~16% in control cells (CTR) to 8% in 7PD rescue cells, while 24PD rescue cells displayed a similar fraction of telomeric TERRA-hTR foci than CTR cells (Fig. 2, F and G). Accordingly, 7PD rescue cells displayed no more than three telomeric TERRA-hTR foci, while up to five TERRA-hTR colocalizations at telomeres were detected in CTR and 24PD rescue samples (Fig. 2H and fig. S2E). During telomere re-lengthening, 7PD rescue cells displayed a larger number of hTR foci and telomeric hTR molecules, compared with 24PD rescue cells (fig. S2, F and G), consistent with the higher telomere elongation rate detected at the early rescue time point. 7PD rescue cells also displayed an increased number of TERRA-hTR foci (fig. S2H) despite showing less TERRA-hTR foci at telomeres (Fig. 2H and fig. S2E), further suggesting a relocalization of TERRA transcripts from telomeres. Consistent with these results, during telomere re-lengthening, we detected a higher number of telomeric hTR molecules not colocalizing with TERRA (TERRA-free telomeric hTR) at 7PD rescue time point compared to 24PD rescue cells (Fig. 2I). No differences in terms of cell cycle profile were observed between samples (fig. S2I). These findings indicate that telomere elongation by telomerase is associated with reduced TERRA localization to chromosome ends, lower number of telomeric TERRA-hTR foci, and increased TERRA-free telomerase molecules at telomeres. This condition may favor telomere re-lengthening by telomerase at the early time point upon termination of BIBR1532 treatment.

### Telomeric TERRA and TERRA-hTR foci decline during telomere elongation in POT1-ΔOB–expressing cells

**Fig. 3.**
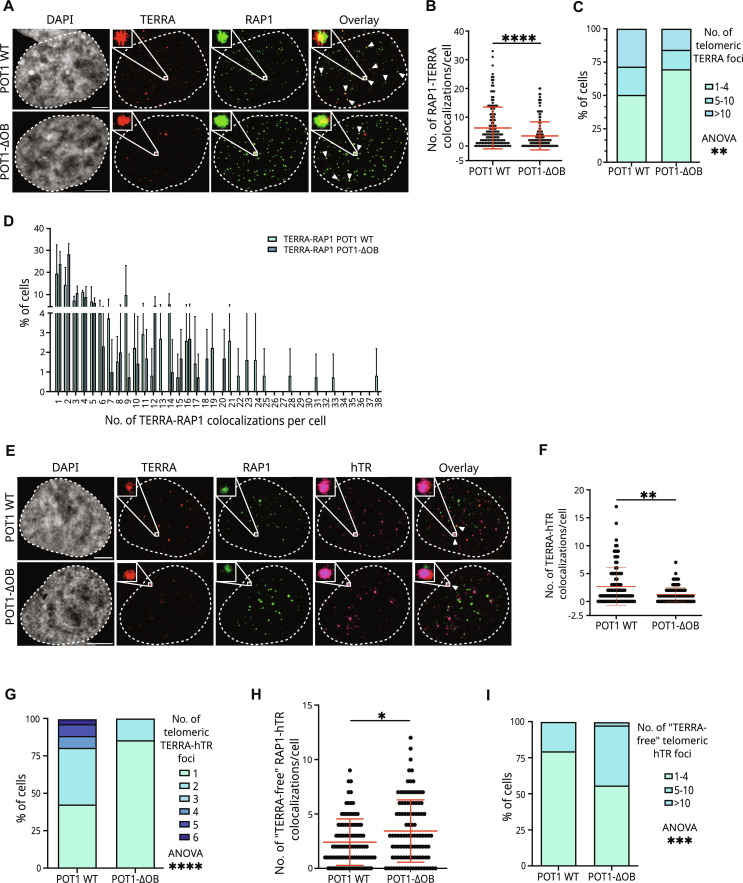
Telomeric TERRA and TERRA-hTR foci decline during telomere elongation in POT1-ΔOB–expressing cells. (**A**) Detection of TERRA and telomeres by smiFISH/IF in HeLa cells expressing POT1 WT or POT1-ΔOB proteins. Scale bar, 5 μm. (**B**) Quantification of the number of telomeric TERRA foci detected per cell by smiFISH/IF (mean ± SD; *n* = 3; 151 POT1 WT cells and 148 POT1-ΔOB cells analyzed). Mann-Whitney test: ****P < 0.0001. (**C** and **D**) Distribution analyses of the number of telomeric TERRA foci per cell. Two-way ANOVA test: **P < 0.01, post hoc Sidak’s multiple-comparison test: *P* = ns. (**E**) Detection of TERRA, hTR, and telomeres by smiFISH/IF in HeLa cells expressing POT1 WT or POT1-ΔOB. Scale bar, 5 μm. (**F**) Quantification of the number of TERRA and hTR colocalizing foci per cell (mean ± SD; *n* = 2; 100 POT1 WT cells and 105 POT1 ΔOB cells analyzed). Mann-Whitney test: **P < 0.01. (**G**) Quantification of the number of telomeric TERRA-hTR foci per nucleus by smiFISH/IF (mean ± SD; *n* = 2; 100 POT1 WT cells and 105 POT1 ΔOB cells analyzed). Two-way ANOVA test: ****P < 0.0001. Post hoc Sidak’s multiple-comparison test: group of one telomeric TERRA-hTR particle per nucleus: *P* = 0.0017, other groups: *P* = ns. (**H**) Number of TERRA-free telomeric hTR foci per cell detected by smiFISH/IF (mean ± SD; *n* = 2; 100 POT1 WT cells and 105 POT1 ΔOB cells analyzed). Mann-Whitney test: *P < 0.05. (**I**) Distribution analyses of the number of TERRA-free telomeric hTR foci from experiments shown in (G). Two-way ANOVA test: ***P < 0.001. Post hoc Sidak’s multiple-comparison test: *P* = ns.

To confirm these results, we expressed a mutant form of the shelterin component POT1 containing a truncation of one of its two OB-fold DNA binding domains (POT1-ΔOB) in HeLa cells. This mutation allows POT1 localization to telomeres but eliminates its ability to bind single-stranded telomeric DNA (*34*). As a result, POT1-ΔOB expression leads to enhanced telomerase retention at telomeres and consequent telomere over-elongation (fig. S3A) (*34*). No major differences in terms of cell cycle profile were detected between cells expressing POT1-ΔOB or POT1 wild type (WT) (fig. S3B). We performed smiFISH/IF experiments to study TERRA localization to telomeres (Fig. 3A). These analyses revealed fewer TERRA foci in POT1-ΔOB cells, compared to POT1 WT cells (fig. S3C). Consistently, lower TERRA levels were detected in POT1-ΔOB cells by RT-qPCR (fig. S3D). These findings are in line with previous evidence indicating low TERRA levels in cells with over-elongated telomeres (*20*). Conversely, a higher number of RAP1 foci were detected in POT1-ΔOB cells, as compared to POT1 WT cells (fig. S3E); integrated density quantification analyses showed that these foci are brighter than the RAP1 foci detected in POT1 WT cells (fig. S3F). These findings are consistent with the presence of longer telomeres in POT1-ΔOB cells (*34*). Quantification of TERRA-RAP1 colocalizations revealed a lower number of telomeric TERRA foci in POT1-ΔOB cells, compared to POT1 WT (Fig. 3B). Consistently, analyses of the distribution of telomeric TERRA foci number showed a lower fraction of cells displaying more than 10 TERRA foci at telomeres in POT1-ΔOB samples, compared to POT1 WT (Fig. 3, C and D). These results indicate that POT1-ΔOB expression correlates with reduced telomeric localization of TERRA in HeLa cells. Notably, for the colocalization analyses performed here, we used the DiAna plug-in of Fiji to confirm significance of the results (details of colocalization analyses are described in Materials and Methods).

Next, we studied TERRA and hTR localizations at telomeres by smiFISH/IF (Fig. 3E). Quantification analyses revealed a lower number of TERRA-hTR foci in POT1-ΔOB cells (Fig. 3F). In these cells, no more than two telomeric TERRA-hTR colocalizations were detected, while up to six telomeric TERRA-hTR foci per cell were observed in POT1 WT cells (Fig. 3G). A higher number of hTR foci and hTR molecules at telomeres were detected in POT1-ΔOB cells (fig. S3, G to I), as expected due to the enhanced telomerase retention at telomeres observed in these cells (*37*). POT1-ΔOB cells displayed a higher number of telomeric hTR molecules not colocalizing with TERRA (TERRA-free telomeric hTR), compared to POT1 WT cells (Fig. 3, H and I, and fig. S3, J and K).

These findings indicate that persistent telomerase retention at telomeres and consequent telomere over-elongation in POT1-ΔOB–expressing cells associate with decreased telomeric localization of TERRA and with a higher number of TERRA-free telomerase molecules at telomeres. This condition may support telomere elongation by telomerase.

### TERRA transcripts preferentially localize at long telomeres in human cells

In *S. cerevisiae*, TERRA transcripts localize preferentially at short telomeres to promote the recruitment of telomerase to these chromosome ends for telomere elongation (*13*, *57*). Our data point to the opposite scenario in human cells, where telomere elongation inversely correlates with TERRA localization at telomeres, suggesting that telomeric TERRA transcripts may interfere with the function of telomerase. Thus, we decided to investigate whether TERRA molecules localize at short or long telomeres in human cells.

To this aim, we used RAP1 IF signal quantification as a proxy for telomere length. To confirm the reliability of this approach, we combined RAP1 IF with telomeric DNA fluorescence in situ hybridization (DNA FISH), a technique that has been extensively used to quantify telomere length (*58*, *59*), but that cannot be easily combined with TERRA smiFISH. By performing telomeric DNA FISH combined with RAP1 IF (DNA FISH/IF), we detected nearly all nuclear RAP1 foci overlapping with the telomeric DNA FISH signal (fig. S4A), confirming, as expected, that RAP1 IF enables the visualization of telomeres at single-cell resolution. Then, we tested whether RAP1 IF signal quantification enables to discriminate telomeres that are longer or shorter than the average telomere length of the cell. To this aim, we performed DNA FISH/IF and estimated the average integrated density of telomeres per cell by quantifying the telomeric DNA FISH signals and RAP1 IF signals. Then, we determined the distance in the integrated density of each telomeric focus from the average integrated density of telomeres of the same cell using both techniques (fig. S4B). These analyses revealed a positive correlation between RAP1 IF and telomeric DNA FISH signals (Spearman’s correlation coefficient = 0.579, *n* = 4378, *P* < 0.0001) (fig. S4C). These findings indicate that integrated density quantification of RAP1 IF foci can be used to discriminate long versus short telomeres compared to the average telomere length of the cell. These results are also in accordance with previous evidence showing that measurements of telomere size by detection of the shelterin protein telomeric repeat-binding factor 1 (TRF1) through IF and telomeric DNA FISH are consistent (*60*).

**Fig. 4.**
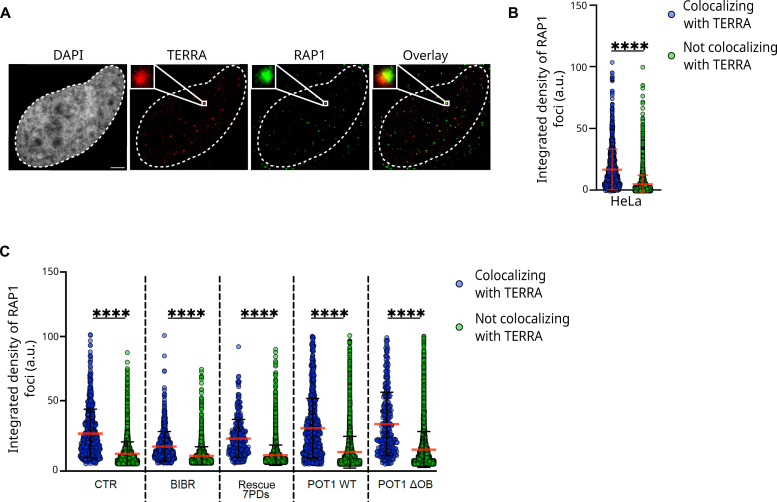
Telomeres colocalizing with TERRA are characterized by high signal intensity as detected by confocal microscopy. (**A**) Detection of TERRA and RAP1 by smiFISH/IF in HeLa cells. An example of a colocalization event between TERRA and RAP1 is shown in the image magnifications. DAPI is used to stain nuclei. Scale bar, 5 μm. (**B**) Integrated density quantification of RAP1 foci colocalizing and not colocalizing with TERRA foci in HeLa cells as detected by smiFISH/IF. Each dot represents a single RAP1 focus. Mean ± SD is shown. A total of 110 cells were analyzed in three independent biological replicates. Statistical significance was assessed by Mann-Whitney test. ****P < 0.0001. (**C**) Integrated density quantification of RAP1 foci colocalizing and not colocalizing with TERRA foci in the indicated samples. Each dot represents a single RAP1 focus. Mean ± SD is shown from the following number of samples and biological replicates: 64 CTR cells (*n* = 2), 67 BIBR1532 cells (123PDs of treatment with BIBR 1532) (*n* = 2), 111 7PD rescue cells (*n* = 2), 151 POT1 WT cells (*n* = 3), and 148 POT1-ΔOB cells (*n* = 3). The Mann-Whitney test was used to assess statistical significance. ****P < 0.0001.

Thus, we performed TERRA smiFISH/RAP1 IF experiments and quantified the integrated density of RAP1 foci colocalizing and not colocalizing with TERRA signal. At these analyses, RAP1 foci colocalizing with TERRA displayed a significantly higher integrated density than RAP1 foci not colocalizing with TERRA (Fig. 4, A and B). We repeated the same analyses on smiFISH/IF experiments performed in cells cultured in different conditions: upon BIBR 1532 (BIBR), or DMSO (CTR), treatment for 123 PDs, at 7PD rescue time point and in cells expressing POT1 WT or POT1-ΔOB. Similar results were observed in all conditions tested, with TERRA-colocalizing RAP1 foci displaying significantly higher integrated density than RAP1 foci not colocalizing with TERRA (Fig. 4C). Furthermore, quantification analyses of TERRA signal revealed higher integrated density of TERRA foci colocalizing with telomeres as compared to the nontelomeric TERRA signal, suggesting that either TERRA clusters or longer TERRA molecules localize to telomeres (fig. S4, D and E).

**Fig. 5.**
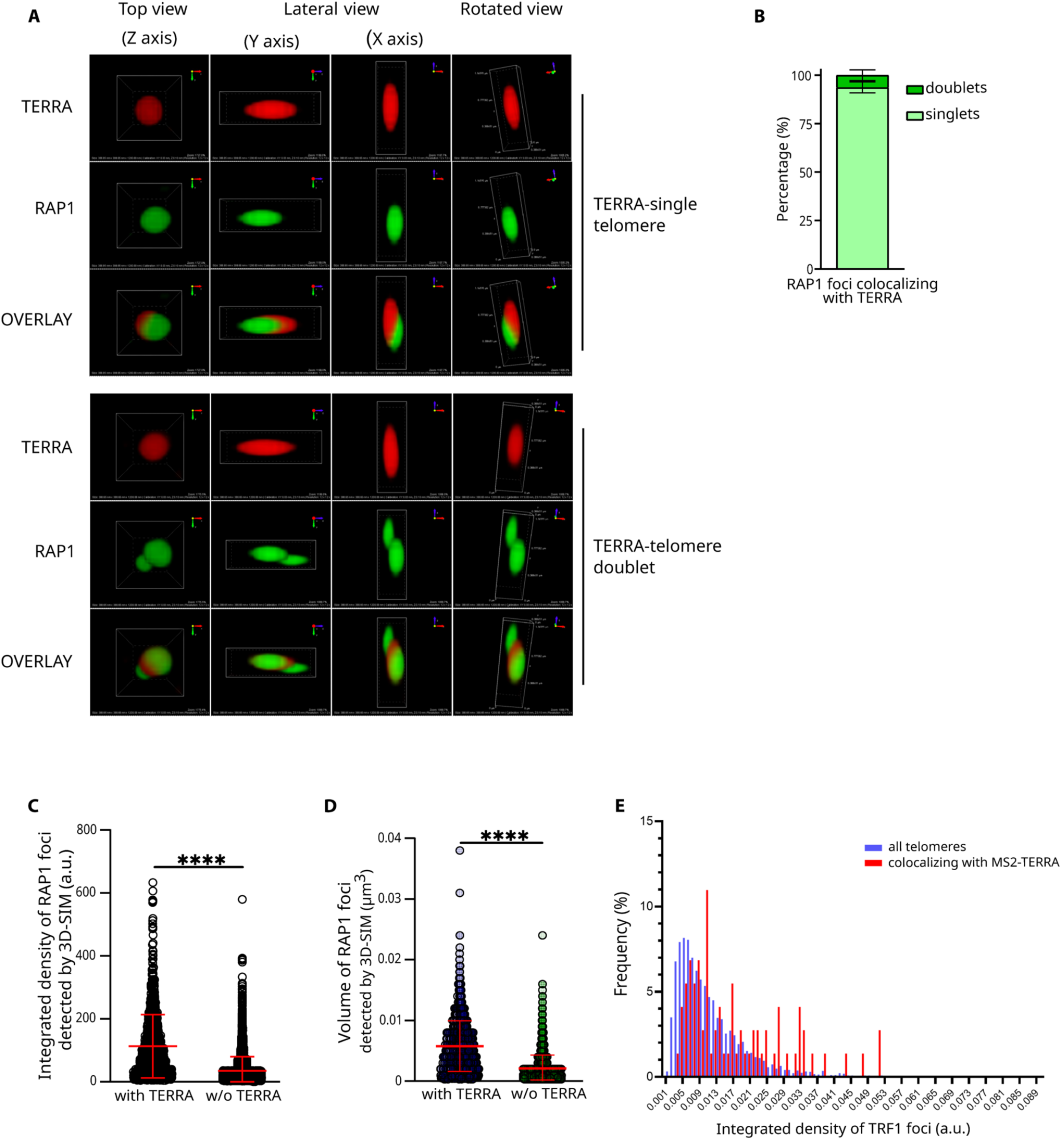
TERRA-associated telomeres are characterized by high signal intensity and larger volume as detected by 3D-SIM microscopy. (**A**) Detection of TERRA and telomeres in HeLa cells by smiFISH/IF. Image acquisitions were performed using three-dimensional structured illumination microscopy (3D-SIM). Examples of TERRA foci colocalizing with a single telomere (top images) or telomere doublet (bottom images) are displayed. TERRA is shown in red; telomeres are in green. Scale bar is indicated in the rotated view images. (**B**) Quantification of the fraction of single telomeres versus telomere doublets colocalizing with TERRA. Data are shown as percentage of TERRA-colocalizing telomeres and represent mean ± SD from three independent biological replicates for a total of 30 cells and 663 TERRA-colocalizing RAP1 foci analyzed. (**C** and **D**) Quantification of the integrated density (C) and volume (D) of RAP1 foci colocalizing (with TERRA) and not colocalizing (without TERRA) with TERRA. Both TERRA-single telomere and TERRA-telomere doublet colocalizations were considered. Data are shown as arbitrary units (a.u.), in (C), and μm^3^, in (D), and represents mean ± SD from three independent biological replicates for a total of 30 cells and 663 TERRA-colocalizing RAP1 foci analyzed. The Mann-Whitney test was used to assess statistical significance. ****P < 0.0001. (**E**) Quantification of the integrated density of all TRF1-mCherry foci and TRF1-mCherry foci colocalizing with MS2-tagged telomere 15q TERRA transcripts per nucleus. Forty nuclei corresponding to 3690 telomeres and 73 telomeres colocalizing with MS2-TERRA transcripts were analyzed from imaging datasets obtained in (*62*). Statistical analysis was performed with a Kolmogorov-Smirnov test: *P* ≤ 0.0001.

To confirm these findings, we repeated the TERRA smiFISH/RAP1 IF experiments imaging cells using three-dimensional structured illumination microscopy (3D-SIM). This super-resolution microscopy technique enabled us to reconstruct images with a resolution of ~115 nm in the lateral (*x*,*y*) direction and ~268 nm in the axial (*z*) direction. Using this approach, we could define the presence of telomere doublets, detected as partially overlapping telomeric foci, which could not be discriminated by diffraction-limited confocal microscopy (Fig. 5A and fig. S5A). In our experiments, approximately 93% of the total number of telomeric foci were detected as single telomeres (fig. S5B). By performing 3D reconstructions of TERRA-RAP1 colocalizations, we observed that more than 90% of TERRA-associated telomeres are singlets (Fig. 5, A and B). TERRA signals colocalizing with telomeres generally overlapped with a major part of the telomere signal, suggesting that TERRA molecules spread over the telomere. Furthermore, we noted that when TERRA colocalized with a telomere doublet, it generally overlapped with the brightest of the two telomeres (Fig. 5A). In line with this observation, quantification analyses revealed that TERRA-colocalizing telomeres retain significantly higher integrated density and volume than telomeres not colocalizing with TERRA (Fig. 5, C and D).

To obtain further evidence in support of these results, we quantified integrated density of telomeres colocalizing with TERRA transcripts expressed from a single telomere in living cells (*61*, *62*). To this aim, we analyzed imaging experiments performed in cells engineered to express endogenous telomere 15q TERRA transcripts tagged with the MS2 bacteriophage sequence (TERRA-MS2 cells). Coexpression of the sfGFP-fused MS2-RNA binding coat protein (MCP-GFP) enabled the visualization of telomere 15q MS2-TERRA molecules in living cells by fluorescence microscopy. Transduction of TERRA-MS2 cells with a retroviral vector expressing TRF1-mCherry to detect telomeres enabled the investigation of TERRA localization to chromosome ends (*61*, *62*). The analyses of live-cell imaging experiments performed in Z-stacks on 40 nuclei revealed that TRF1-mCherry foci colocalizing with telomere 15q MS2-TERRA molecules display a significantly higher integrated density compared to the average TRF1-mCherry signal of the same cell (Fig. 5E) (*P* < 0.0001). These data are consistent with the results obtained by smiFISH/IF experiments.

Together, these results indicate that TERRA transcripts preferentially localize at long telomeres in human cells. This condition is independent on the average telomere length of the cell as it is observed in cells harboring short (BIBR1532-treated cells) as well as long telomeres (POT1-ΔOB–expressing cells).

### Human TERRA transcripts localize at chromosome ends in trans

Previous evidence indicates that human cells with short telomeres express higher TERRA levels than cells with longer telomeres, suggesting that TERRA expression is up-regulated when chromosome ends are short (*20*, *63*). Since we detected TERRA transcripts preferentially at long telomeres, we asked whether TERRA localizes to its telomere of origin or relocate to different chromosome ends.

To address this question and study the localization of TERRA with respect to its telomere of origin, we used the clustered regularly interspaced palindromic repeats (CRISPR)/catalytically dead CRISPR-associated protein 9 (dCas9) (CRISPR/dCas9) reporter system to label the subtelomeric region of chromosome 15q in TERRA-MS2 cells. The use of the CRISPR/dCas9 system has revealed a robust approach to detect human genomic loci by live-cell imaging (*64*). We designed a small guide RNA (sgRNA) targeting a sequence repeated 71 times within subtelomere 15q. Coexpression of this sgRNA with the dCas9-BFP fusion protein enabled the detection of two discrete dCas9-BFP nuclear foci per cell, which colocalized with the TRF1-mCherry signal (fig. S6A), in line with the presence of two copies of chromosome 15q in the AGS cell line (*65*).

**Fig. 6.**
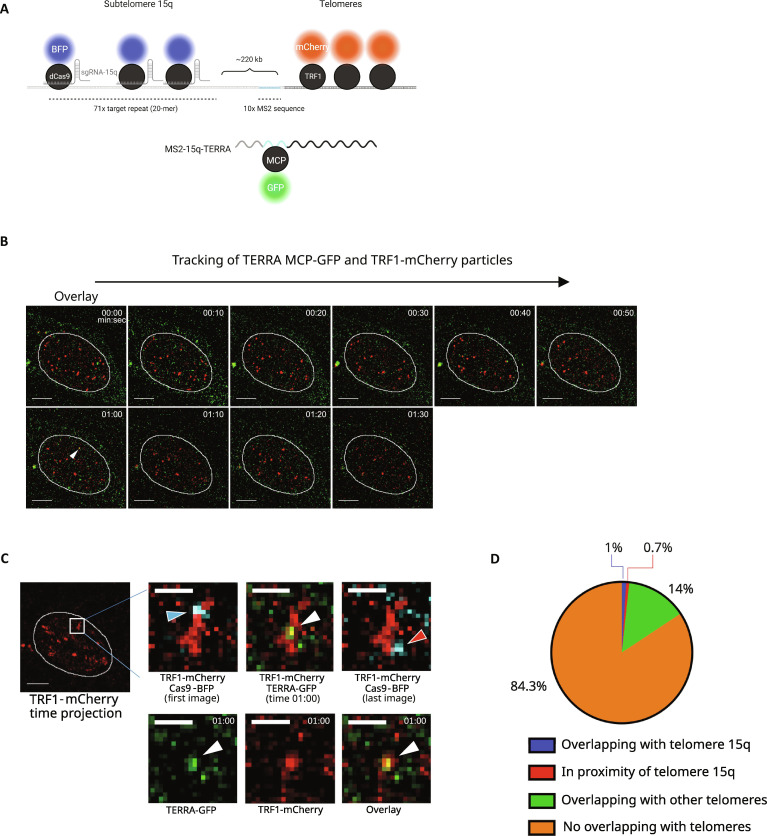
Human TERRA transcripts localize at chromosome ends in trans. (**A**) Live-cell imaging approaches used to simultaneously detect telomere 15q TERRA transcripts, subtelomere 15q, and telomeres. 10xMS2 sequences were integrated within subtelomere 15q. Telomere 15q MS2-TERRA is detected by MCP-sfGFP expression. Subtelomere 15q is visualized by expression of dCas9-BFP and sgRNA targeting the subtelomere. Telomeres are visualized by TRF1-mCherry expression. (**B**) Representative images of a time-lapse experiment performed to track TERRA MCP-sfGFP and telomeres. TERRA-telomere colocalizations were analyzed by two-color Z-stack acquisitions every 10 s. dCas9-BFP signal was imaged at the first and last time points. TERRA MCP-sfGFP particles are in green, and telomeres are in red; a single Z-slice is shown. The white arrowhead at the 1-min time point (1:00) indicates a colocalization event between TERRA MCP-sfGFP and a telomeric signal corresponding to telomere 15q. Scale bars, 5 μm. (**C**) Verification of the colocalization between telomere 15q TERRA (TERRA-GFP) and its telomere of origin. A maximum intensity projection of all the TRF1-mCherry images of the time lapse for a single Z-slice was performed to show the trajectory of telomere 15q, overlaid with the dCas9-BFP foci at the first (blue arrow) and last (red arrow) time points of the experiment. At the 1-min time point, the TERRA MCP-sfGFP spot (white arrow) colocalizes with telomere 15q. Deconvolution was performed with AutoQuant software. Scale bars, 5 and 1.2 μm for the zoom. (**D**) Quantification of the number of TERRA MCP-sfGFP particles colocalizing with their telomere of origin or other telomeres. Percentage of TERRA MCP-sfGFP particles colocalizing with telomere 15q (blue), detected in proximity of telomere 15q (red) (0.6 μm distance), overlapping with other telomeres (green) or not colocalizing with, and not in proximity of, telomeres (orange) is shown. Twenty-two cells were analyzed for a total of 408 TERRA MCP-GFP particles tracked.

To test whether TERRA molecules localize at their telomere of origin, we performed live imaging of MCP-GFP–expressing TERRA-MS2 cells transduced with a retroviral vector expressing TRF1-mCherry to detect telomeres, and cotransfected with vectors expressing dCas9-BFP and the subtelomere 15q-targeting sgRNA (Fig. 6A). We performed TERRA MCP-GFP and TRF1-mCherry imaging in time lapse using a spinning disk confocal microscope (Fig. 6B and movies S1 and S2). The colocalization between the TRF1-mCherry and dCas9-BFP signals enabled us to identify telomere 15q (Fig. 6C). By tracking 408 TERRA MCP-GFP particles with the use of the TrackMate algorithm (*66*) (fig. S6B), we observed 4 particles colocalizing with telomere 15q and 3 particles localizing to its proximity (<0.6 μm), for a total of 7 of 408 particles localizing in cis at their telomere of origin (1.7%) (Fig. 6D). Fifty-seven particles were detected at other telomeres (14%), while 344 of 408 did not localize at telomeres (84.3%) (Fig. 6D).

These findings indicate that TERRA transcripts are mostly nontelomeric and they interact with telomeres transiently. TERRA predominantly localizes at telomeres in trans by relocating to chromosome ends, which are different from their transcription site.

### Depletion of TERRA results in increased telomerase RNA clustering and localization to telomeres

**Fig. 7.**
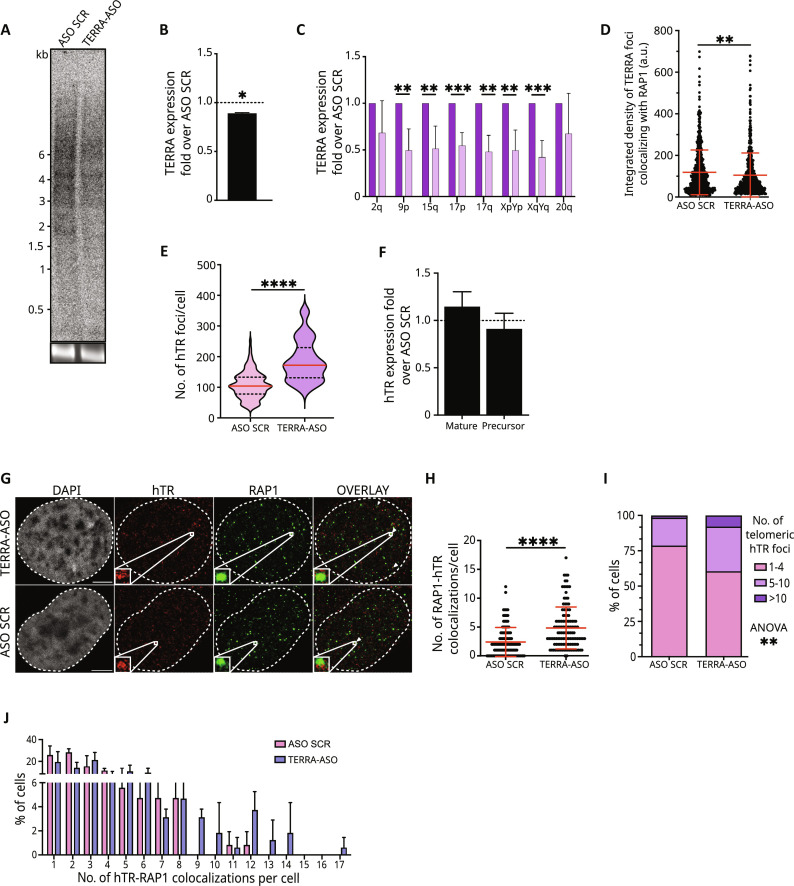
TERRA depletion results in increased hTR clustering and localization at telomeres. (**A**) Northern blot analysis of TERRA in HeLa cells upon TERRA-ASO or control ASO (ASO SCR) transfection. Bottom image shows 18*S* rRNA band upon gel run. (**B**) Quantification of TERRA signal from Northern blot analyses of TERRA-ASO–transfected cells shown as fold over ASO SCR (dashed line). *P < 0.05; mean ± SD, *n* = 2. (**C**) RT-qPCR analyses of TERRA expression from the indicated telomeres in TERRA-ASO cells shown as fold over ASO SCR. Mean ± SD from four independent biological replicates. Unpaired *t* test: **P < 0.01, ***P < 0.001. (**D**) Integrated density quantification of TERRA foci colocalizing with RAP1 foci. Each dot represents a single TERRA signal (mean ± SD; *n* = 2; 131 ASO SCR cells and 122 TERRA-ASO cells analyzed). Mann-Whitney test: **P < 0.01. (**E**) Quantification of the number of hTR foci detected per nucleus in TERRA-ASO and ASO SCR cells (mean ± SD; *n* = 2; 131 ASO SCR cells and 122 TERRA-ASO cells analyzed). Mann-Whitney test: ****P < 0.0001. (**F**) RT-qPCR quantification of hTR levels using primer pairs detecting the precursor or mature RNA (*78*, *79*). Results are shown as fold change over ASO SCR (dashed line) (mean ± SD, *n* = 2). U6 gene was used for normalization (*80*). (**G**) Detection of hTR and telomeres by smiFISH/IF. Scale bar, 5 μm. (**H**) Quantification of the number of telomeric hTR foci detected per nucleus. Data are shown as number of RAP1-hTR colocalizations per cell (each dot represents a cell) (mean ± SD, *n* = 2; 131 ASO SCR cells and 122 TERRA-ASO cells analyzed). Mann-Whitney test: ****P < 0.0001. (**I** and **J**) Distribution analysis of the number of RAP1-hTR colocalizations detected per cell. Two-way ANOVA test: **P < 0.01.

Our results indicate that TERRA localization to chromosome ends declines during telomere elongation by telomerase, a process associated with a decreased number of telomeric TERRA-hTR foci. Furthermore, when interacting with telomeres, TERRA molecules preferentially localize to long chromosome ends, which are expected to be less frequently elongated by telomerase than short telomeres. Therefore, we formulated the hypothesis that TERRA molecules may interfere with telomerase function by impairing its recruitment and/or retention to chromosome ends. To investigate this possibility, we used ASOs targeting the telomeric repeat tract of TERRA (TERRA-ASO) to down-regulate TERRA molecules in cells and investigated the impact on telomerase localization to chromosome ends. TERRA-ASO transfection led to depletion of TERRA levels as detected by Northern blot (Fig. 7, A and B) and RT-qPCR (Fig. 7C), in accordance with previous evidence obtained in different cell types (*43*, *67*, *68*). Furthermore, TERRA-ASO–transfected cells displayed less intense telomeric TERRA foci, compared to control cells transfected with ASO scrambled (ASO SCR), suggesting that a lower number of TERRA molecules associate with telomeres (Fig. 7D). TERRA down-regulation did not lead to significant telomere damage or replicative stress at chromosome ends, as assessed by the number of telomere dysfunction–induced foci (TIF) and RPA32 foci colocalizing with telomeres (fig. S7, A and B). smiFISH experiments revealed an increased number of hTR foci per nucleus in TERRA-ASO–transfected cells (Fig. 7E). This result is consistent with previous evidence showing a higher number of TR foci in mESCs upon TERRA depletion by ASO (*43*). Differently from the condition observed in mESCs, RT-qPCR analyses revealed no differences in hTR transcript levels upon TERRA down-regulation in HeLa cells (Fig. 7F). These findings suggest that TERRA depletion influences hTR transcript clustering. We analyzed telomerase localization to telomeres by performing hTR smiFISH/RAP1 IF experiments in TERRA-ASO– and ASO SCR–transfected cells (Fig. 7G). Quantification analyses revealed a significant increase in the number of telomeric hTR foci in TERRA-depleted cells (Fig. 7H). In line with these findings, we detected a higher number of cells displaying more than 10 telomeric hTR foci in TERRA-ASO–transfected samples, compared to ASO SCR samples (Fig. 7, I and J). These results indicate that TERRA levels influence hTR foci formation and their localization at telomeres, suggesting that TERRA impedes hTR clustering and its recruitment and/or retention at chromosome ends.

### Depletion of TERRA leads to increased telomerase RNA retention at telomeres

**Fig. 8.**
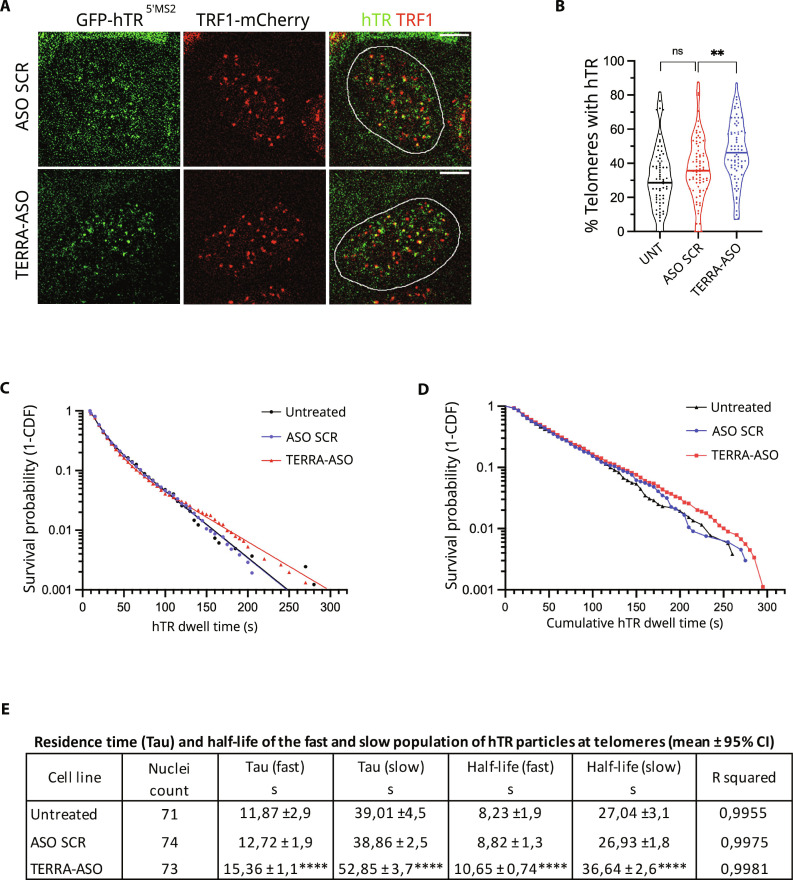
TERRA depletion increases hTR retention at telomeres. (**A**) HeLa hTR^5’MS2^ + hTERT cells were transfected with TERRA-ASO, ASO SCR, or the transfection reagent (UNT) for 48 hours. Still images of telomeres (in red) visualized with TRF1-mCherry and hTR (in green) bound by MCP-sfGFP. Colocalization events are in yellow. Nuclear outline is in white. Scale bar, 5 μm. (**B**) Percentage of telomeres colocalized with hTR, defined as telomeres that had at least one stable colocalized hTR track, lasting 10 s or more. TERRA-ASO versus ASO SCR, **P = 0.02; UNT versus ASO SCR *t* test shows nonsignificant difference; *n* = 71 to 74 cells per condition. (**C**) Survival probability analysis of individual hTR particles at telomeres in HeLa hTR^5’MS2^ + hTERT cells transfected with TERRA-ASO, ASO SCR, or the transfection reagent (UNT) for 48 hours. (**D**) Survival probability analysis of the cumulative dwell time of hTR particles at individual telomeres in two independent replicates. Median values: untreated: 40 s, ASO SCR: 40 s, and TERRA-ASO: 45 s. Statistical analysis with log-rank test: 0.296 (ns). (**E**) Fast (probing) and slow (binding) values for hTR residence time (Tau) and half-life obtained from bi-exponential decay curves in (C). Statistical analysis between ASO SCR and TERRA-ASO using multiple unpaired *t* test: ****P < 0.000001; statistical analysis between untreated versus ASO SCR using multiple unpaired *t* test: ns.

To gain further insight into the mechanism by which TERRA regulates hTR localization at telomeres, we used a HeLa cell line expressing MS2-tagged endogenous hTR molecules and the sfGFP-fused MS2-RNA binding coat protein (MCP-sfGFP) recently developed to study telomerase dynamics at telomeres in living cells by fluorescence microscopy (*37*). Using this cellular model, we performed hTR single-particle and telomere tracking by dual imaging in time lapse of hTR molecules (GFP-hTR^5’MS2^) and telomeres (TRF1-mCherry), upon TERRA down-regulation by ASO transfection, and in control cells (Fig. 8A). Quantification analyses revealed an increase in the percentage of telomeres colocalizing with hTR, from 36% in control cells to 46% in TERRA-depleted cells (Fig. 8B). Furthermore, by fitting the dwell time of hTR molecules at telomeres in a survival probability plot (Fig. 8, C and D), we measured a significant increase in the residence time and half-life of both fast-moving and slow-diffusing hTR particles at telomeres in TERRA-depleted cells (Fig. 8E and movies S3 to S6). These results indicate that TERRA depletion associates with increased telomerase RNA retention at telomeres, suggesting that TERRA transcripts can regulate telomere elongation by inhibiting telomerase access to chromosome ends.

## DISCUSSION

Here, we investigated the role of TERRA in the regulation of telomerase in human cancer cells. To date, this function of TERRA transcripts has been difficult to define (*69*). TERRA interaction with telomerase is mediated at least in part through the binding between the telomeric tract of TERRA and the template region of hTR (*41*). This interaction impairs telomerase activity in vitro, suggesting that TERRA molecules act as inhibitors of telomerase. However, somewhat challenging this model, evidence in vivo indicates that increased TERRA expression does not interfere with telomere elongation by telomerase, suggesting that mechanisms are in place to prevent telomerase inhibition by TERRA (*44*–*46*). To us, a missing piece in this riddle was the information on where in the nucleus and when during the cell cycle TERRA-telomerase interaction may occur.

Our experiments of single-molecule RNA fluorescence in situ hybridization indicate that TERRA and telomerase can interact both in the nucleoplasm and at telomeres. TERRA-hTR foci were detected at telomeres also during S phase. This is notable because telomere elongation takes place during this phase of the cell cycle. Recently, key advances into the dynamics of telomerase recruitment to telomeres came from single-molecule live-cell imaging. These studies using different approaches to track hTERT or hTR molecules revealed that only a few telomeres are elongated by telomerase at any given time in S phase. It was reported that while human telomerase transiently localizes to multiple chromosome ends in nonproductive associations mediated by TPP1-hTERT interaction, it stably interacts with only a few telomeres to carry out their elongation (*37*, *39*).

Our data suggest that telomeres displaying TERRA-hTR foci are not actively elongated by telomerase. We analyzed TERRA and hTR localizations during telomere elongation, by inducing telomere shortening through transient telomerase inhibition and then allowing telomere lengthening upon removal of the inhibitor. This resulted in a transient enhancement in telomere elongation at the first time point (7PD rescue), after which telomere lengthening attenuated as chromosome ends became longer. In these experiments, 7PD rescue cells displayed a significant decrease in the number of telomeric TERRA-hTR foci. Our analyses suggest that this is due to a relocalization of TERRA molecules away from chromosome ends, indicating that TERRA at telomeres may interfere with their elongation by telomerase. This scenario was confirmed using a different approach, in which telomere elongation was promoted through expression of the POT1-ΔOB mutant protein, leading to enhanced telomerase retention at chromosome ends and consequently telomere over-elongation. Also in this condition, we observed impaired TERRA localization at telomeres and decreased number of telomeric TERRA-hTR foci. Notably, 7PD rescue cells and POT1-ΔOB–expressing cells have very different average telomere lengths, estimated to 2.75 kb and more than 10 kb, respectively. Thus, TERRA relocalization from chromosome ends and decreased TERRA-hTR foci at telomeres occur during telomere elongation independently from the overall telomere length of the cell.

Telomere elongation by telomerase requires base pairing of the template region of hTR with the G-rich 3′ overhang of the telomere (*31*). Furthermore, it has been recently shown that hTR base pairing with the telomeric 3′ overhang is required to promote telomerase retention to the end of chromosomes (*37*). As the 3′ end of TERRA can base pair with the hTR template sequence, it is conceivable that telomeric TERRA transcripts may interfere with telomere elongation by preventing hTR pairing with chromosome ends. Furthermore, TERRA molecules may compete with hTR engaged at the 3′ overhang and accelerate its dissociation. To gain insight into the mechanism of TERRA-mediated telomerase regulation, we depleted TERRA using ASOs and studied telomerase localization to telomeres. Decreased TERRA levels and consequent lower number of TERRA molecules at telomeres resulted in an increased number of telomeric hTR foci. By tracking hTR single particles in living cells, we found that TERRA depletion also results in increased half-life and extended residence time of hTR particles at telomeres. This condition may result at least in part from increased access of telomeric hTR to the single-stranded 3′ overhang of telomeres and/or decreased dissociation of hTR molecules engaged at the 3′ overhang, supporting a scenario in which telomeric TERRA transcripts interfere with hTR base pairing with chromosome ends. In a recent study, single-particle tracking of hTR revealed a two-step recruitment-retention model of telomere elongation by telomerase whereby TPP1-mediated recruitment of telomerase leads to short and highly diffusive telomerase-telomere interactions, while retention of telomerase to telomeres, mediated by base pairing of hTR template sequence with the single-strand telomeric overhang, results in long-lasting hTR-telomere interactions, longer hTR residence time at telomeres, and consequent telomere elongation (*37*). A similar behavior of telomerase was reported by live-cell imaging of Halo-hTERT (*39*). In light of these findings, our results support a model by which telomeric TERRA transcripts regulate telomere elongation by interfering with telomerase retention at chromosome ends. Unexpectedly, TERRA depletion resulted in an increased number of hTR foci despite its levels remaining unchanged. Considering these results, it is intriguing to hypothesize that TERRA-hTR interactions may influence hTR clustering. Future investigations will be directed toward this potential function of TERRA.

Live-cell imaging, confocal microscopy, and 3D-SIM super-resolution microscopy revealed that telomeric TERRA transcripts preferentially localize at long telomeres, regardless of the overall average telomere length of the cells. For the average telomere length to be maintained, longer telomeres are expected to be less prone to telomerase-mediated elongation compared to the shortest telomeres. This mechanism, which is mediated by the increased amount of telomerase negative regulators at long telomeres, has been previously described in yeast (*70*), and it may occur also in human cells (*71*). Our results indicating most prominent telomere elongation at 7PD rescue time point, compared to 24PD and 45PD rescue time points, are in accordance with this model. We propose that the presence of TERRA at long telomeres may contribute to the inhibition of telomerase at these chromosome ends. TERRA recruitment to telomeres can be mediated by the interaction with telomeric proteins, including the shelterin proteins TRF1 and TRF2 (*11*), or by formation of R-loop structures within the telomeric repeat tract (*14*). Therefore, longer telomeres are expected to have a higher number of “docking sites” for TERRA. Accordingly, we noted that TERRA transcripts can form clusters at telomeres, as indicated by quantification analyses of TERRA signal from smiFISH experiments revealing higher integrated density of telomeric TERRA foci, compared to the extratelomeric TERRA signal. Notably, these results are consistent with previous evidence indicating a correlation between intensities of TERRA and colocalizing telomeric foci in human cells (*20*). An alternative interpretation of these results is that telomeres interact with TERRA molecules that have longer telomeric repeat sequences than the transcripts present at extratelomeric sites. As the TERRA probe binds the repetitive 3′ end sequence of TERRA, longer 3′ ends of the transcripts will result in more probes bound to the RNA and consequent increase in signal intensity. Furthermore, it may be possible that telomeric TERRA transcripts retain structural features, such as less G4 structures, rendering them more accessible to the probe. Either of these conditions is expected to promote the base pairing of TERRA with hTR. In this regard, imaging of TERRA-telomere colocalizations by 3D-SIM also revealed that TERRA transcripts can cover a major part of the telomere. This condition may create a “telomerase repressive” environment at these chromosome ends.

**Fig. 9.**
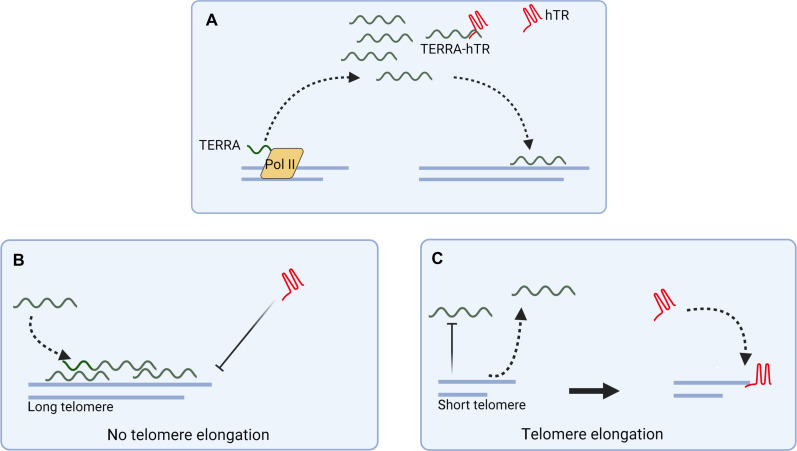
Proposed model. TERRA transcripts are displaced from their telomere of origin to localize within the nucleoplasm, associating or not with hTR. (**A**) A fraction of nucleoplasmic TERRA molecules is recruited at long telomeres. (**B**) Accumulation of TERRA at long telomeres interferes with telomerase localization and retention at these chromosome ends. Short telomeres either displace TERRA molecules or do not attract TERRA. (**C**) Telomerase is recruited at these chromosome ends to promote telomere elongation. Created with Biorender.com

Multiple functions have been proposed for TERRA at chromosome ends, including chromatin formation, assisting DNA replication, and telomere capping (*11*, *20*–*22*). How these functions are coordinated perhaps at distinct telomeres remains unknown. A key element in this process is represented by the complex dynamics of these transcripts. The telomeric localization of TERRA is tightly controlled, and multiple mechanisms have been identified to actively displace TERRA molecules from telomeres (*1*, *10*). By using a combination of live-cell imaging approaches to simultaneously visualize TERRA transcripts expressed from a single telomere, their telomere of origin and chromosome ends, we were able to track TERRA particles and study their dynamics with respect to telomeres in time-lapse experiments. In these analyses, we observed that TERRA molecules preferentially localize at telomeres in trans, by relocating from their transcription site. Furthermore, we found that TERRA particles mainly reside outside telomeres, confirming that TERRA localization to telomeres is highly dynamic. Notably, it was recently shown that chimeric TERRA transcripts expressed from an intrachromosomal locus are able to localize to telomeres, indicative of their capability to relocate away from their transcription site (*14*). Here, it was observed that HeLa clones with short telomeres display increased telomeric localization of the chimeric TERRA transcripts compared to HeLa clones containing long telomeres. It will be interesting to investigate whether TERRA localizes at long telomeres also in these clones. Our results suggest that even in cells with short telomeres TERRA preferentially localizes to the longer chromosome ends. Overall, our findings support a scenario in which upon transcription, TERRA molecules are displaced from their telomere of origin to localize within the nucleoplasm (Fig. 9). A fraction of this pool of TERRA transcripts interacts with hTR. Long telomeres can attract TERRA molecules due to the high number of TERRA-interacting proteins bound to, and/or the high number of telomeric repeats present at, these chromosome ends. At these telomeres, TERRA transcripts interfere with telomerase retention by base pairing with hTR. Short telomeres do not attract or displace TERRA transcripts, a mechanism that contributes to telomerase retention at these chromosome ends. This model enables us to reconcile at least in part the conflicting evidence obtained in vivo and in vitro on the function of TERRA in the regulation of telomerase. TERRA can act as repressor of telomerase as indicated by in vitro approaches (*2*, *41*, *42*), yet increased TERRA transcription will not interfere with telomere elongation in vivo, as observed during generation of iPSCs (*45*–*49*), since the transcripts can be displaced from telomeres. Our observations that TERRA molecules localize in trans, at chromosome ends that are different from their telomere of origin, can also help explain why TERRA expression from an inducible promoter integrated at a single chromosome end does not impair telomerase activity in cis (*44*).

Notably, telomerase distribution to telomeres is regulated by TERRA transcripts also in budding yeast (*13*). In this species, TERRA molecules are displaced from their telomere of origin upon transcription, and a fraction of the nucleoplasmic pool of TERRA interacts with the telomerase RNA subunit TLC1. This scenario resembles our observations in human cells. However, it was reported that TERRA-TLC1 interaction promotes telomerase RNA clustering, thereby favoring telomerase recruitment to the short telomere found to induce TERRA expression (*13*). Conversely, our results indicate that TERRA can interfere with telomerase localization at telomeres in human cells and it may also impair hTR clustering. Thus, the function of TERRA in the regulation of telomerase is not conserved between these two species, and while TERRA positively regulates telomerase in budding yeast, it can repress telomerase in humans.

Previous evidence indicates that TERRA positively regulates telomerase also in fission yeast (*40*). In this organism, telomerase regulation is mediated by polyadenylated TERRA transcripts. In future studies, it will be interesting to investigate whether poly(A)^+^ and poly(A)^−^ TERRA transcripts are differently involved in regulating telomerase in human cells. Our findings highlight the importance of investigating TERRA in different model systems to obtain a complete picture of the diverse functions that this RNA can mediate in cells.

### Limitations of the study

Here, we focused on understanding the dynamics of TERRA and TERRA-hTR molecules during telomere elongation, providing evidence of the function of TERRA in the regulation of human telomerase. A limitation of the study is that it did not identify the factors that displace TERRA molecules from telomeres during telomere lengthening. Several TERRA-binding proteins may be involved in this mechanism. In future studies, it will be important to also investigate whether poly(A)^+^ and poly(A)^−^ TERRA transcripts are differently involved in telomerase regulation in human cells.

## MATERIALS AND METHODS

### Cell culture

HeLa, human embryonic kidney (HEK) 293T, HEK 293 Phoenix, and HCT116 cell lines were cultured in Dulbecco’s Modified Eagle’s medium (DMEM) (Gibco) supplemented with 10% fetal bovine serum (Gibco), 2 mM l-glutamine (Gibco), and penicillin-streptomycin solution (100 U/ml) (Gibco). TERRA-MS2 cells were previously generated in the AGS human stomach cancer cell line (*61*, *62*) and cultured in DMEM supplemented with 10% fetal bovine serum (Gibco), 2 mM Glutamax (Gibco), and penicillin-streptomycin solution (100 U/ml) (Gibco). Cells were incubated at 37°C with 5% CO_2_, passaged every 48 to 72 hours, and regularly tested for mycoplasma contamination. Treatment with the BIBR1532 compound (Cayman Chemical) was performed by culturing cells for the indicated generations in complete medium containing BIBR1532 (10 μM final concentration). BIBR1532-supplemented medium was freshly added to cells every 48 to 72 hours or during splitting. Control cells (CTR) were cultured in complete medium containing DMSO for the same period. POT1 WT and POT1-ΔOB–expressing cells were selected by transduction of HeLa cells with retroviral vector particles generated using pLPC myc-hPOT1 and pLPC myc-hPOT1-ΔOB plasmids, as described in the dedicated materials and methods section. Cells were selected in puromycin-containing DMEM (0.7 μg/ml of puromycin) for 1 week.

### Cell synchronization

Cells were synchronized using double thymidine block as previously described in literature (*72*). Cells at 40% confluence were cultured in thymidine-containing DMEM (2 mM final concentration) for 18 hours at 37°C. After washing with 1× phosphate-buffered saline (PBS), cells were cultured in fresh medium for 8 hours, and then a second incubation in thymidine-containing DMEM (2 mM final concentration) for 18 hours at 37°C was performed. S-, G_2_-, and G_1_-phase cells were collected at 4, 10, and 16 hours after release from the second thymidine incubation.

### Generation of viral particles and cell transduction

Retroviral and lentiviral vector particles were produced using HEK 293 Phoenix and HEK 293T cells, respectively. Cells were seeded in antibiotic-free medium and transfected with the required plasmids using calcium phosphate or jetPRIME transfection reagent (Polyplus). A list of the plasmids used in this study is provided in table S1 (*73*). For retroviral vector particle generation used for POT1 WT and POT1-ΔOB expression, 24 hours after plasmid transfection, Phoenix cells were treated with 10 mM sodium butyrate (Sigma-Aldrich) for 8 hours. Supernatants were harvested 48 hours from transfection, filtered using 0.22-μm Primo Syringe Filters (Euroclone), and precipitated overnight in PEG-8000 (polyethylene glycol, molecular weight 8000) (Sigma-Aldrich). The following day, the viral vector particles were centrifuged for 15 min at 2000*g* and resuspended in medium without serum. Aliquots of viral vector particles were stored at −80°C. For the selection of POT1 WT and POT1-ΔOB–expressing cells, HeLa cells were transduced at 50 to 60% confluence, by replacing the culturing medium with fresh DMEM containing retroviral vector particles and polybrene (8 μg/ml). Twenty-four hours later, polybrene-containing medium was replaced with fresh medium. Forty-eight hours post transduction, cells were selected in puromycin-containing medium (0.7 μg/ml of puromycin) for 1 week before performing experiments.

For lentiviral vector particle generation required for expression of MCP-sfGFP and TRF1-mCherry fusion proteins, HEK 293T cells were transfected with appropriate plasmids using jetPRIME following the manufacturer’s instructions. The culturing medium was changed after 24 hours from transfection. Forty-eight hours after transfection, supernatant was filtered with a polyethersulfone (PES) 0.45-μm syringe filter. Supernatant was freshly used or aliquoted in 1.5-ml tubes, flash-frozen, and kept at −80°C. Transduction of lentiviral vector particles was performed on cells cultured to 50 to 60% confluence.

### Single-molecule inexpensive RNA fluorescence in situ hybridization

The smiFISH protocol was adapted from Querido *et al.* (*52*). Cells were grown on 22 × 22 mm glass coverslips (Prestige) previously stripped with 1 M HCl, sterilized, and stored in 100% ethanol. Cell fixation was performed in 4% paraformaldehyde (PFA) (Electron Microscopy Science) for 20 min at room temperature. After washing two times with 1× PBS, cells were kept at +4°C for maximum 1 month or directly permeabilized in 0.5% Triton X-100, 1× PBS for 5 min at room temperature. Control samples were treated with RNase T1 (300 U/ml) (Macherey-Nagel) and PureLink RNase A (200 μg/ml) (Invitrogen) for at least 2 hours at 37°C. Once permeabilized, samples were incubated at room temperature in a solution containing 1× SSC and 15% formamide, for 40 min and placed cell-side down on a 50-μl drop of hybridization buffer containing the probe [1× SSC, 15% formamide, bovine serum albumin (BSA) (4.5 mg/ml), 10.6% dextran sulfate, 2 mM Vanadyl Ribonucleoside Complex (VRC), yeast tRNA (0.4 mg/ml), 1 μl of probe mix] in an air-tight hybridization chamber protected from light overnight at 37°C. The probe mix preparation is described in the next paragraph. The following day, coverslips were washed twice with prewarmed 1× SSC solution containing 15% formamide for 30 min at 37°C. After two rinses with 1× PBS, cells were stained with DAPI (1 ng/μl) (4′,6-diamidino-2-phenylindole) in 1× PBS, for 8 min at room temperature and washed with 1× PBS. Samples were mounted on precleaned glass slides using ProLong Diamond Antifade Mountant (Thermo Fisher Scientific).

### smiFISH probe preparation

Probe preparation was performed as described by Querido *et al*. (*52*). For hTR detection 15 primary probes targeting the hTR sequence were used. Sequences of the probes used in smiFISH experiments are shown in table S2. The hTR primary probeset was prepared as a 20 μM equimolar mixture (1.33 μM, or 40 pmol of each probe) in milliQ water. For TERRA detection, a single primary probe was used at 20 μM concentration in milliQ water. Before hybridization, primary probes were mixed with 50 pmol of fluorescently labeled secondary probes, specific for TERRA primary probe or hTR primary probeset, in 1× NEB3 solution [100 mM NaCl, 50 mM tris-HCl (pH 8), 10 mM MgCl_2_] in a final volume of 10 μl. Primary probes were annealed with secondary probes in a PCR thermocycler (85°C for 3 min, 65°C for 3 min, 25°C for 5 min). Dual-color smiFISH was performed by annealing each specific primary probe with its secondary probe in a separate tube and, once annealed, mixed together in the hybridization buffer. The probe mix (1 μl) was used for each smiFISH experiment as described in the previous paragraph.

### Immunofluorescence

Cells were seeded on 22 × 22 mm glass coverslips (Prestige) previously stripped with 1 M HCl, sterilized, and stored in 100% ethanol. Cells were fixed in 1× PBS, 4% PFA (Electron Microscopy Science) for 20 min at room temperature. After fixation, cells were washed twice in 1× PBS and kept at +4°C for maximum 1 month or directly permeabilized in 1× PBS, 0.5% Triton X-100, for 5 min at room temperature. After two rinses in 1× PBS, cells were incubated in blocking solution (1× PBS, 2% BSA, 0.1% Triton X-100) for 3 hours and incubated for 1 hour at room temperature with the primary antibody diluted in blocking solution. For RAP1 detection, we used the rabbit anti–TERF2-IP (immunoprecipitation) antibody, from Novus Biological (catalog no. NB100-292; RRID: AB_10000825), at 1:500 dilution. For γH2AX detection, we used the mouse monoclonal anti–phospho–histone H2A.X, from Millipore (catalog no. 05-636; RRID: AB_309864), at 1:500 dilution. For RPA32 detection, we used mouse monoclonal anti-RPA32 (9H8) from Abcam (catalog no. ab2175), at 1:350 dilution. Cells were then washed six times for 5 min with 1× PBS, 0.1% Triton X-100 and incubated with the appropriate fluorescent secondary antibody for 1 hour [goat anti-rabbit Alexa Fluor 488 (AF488), from Thermo Fisher Scientific, catalog no. A-11034, 1:1500 dilution; donkey anti-mouse AF647, from Thermo Fisher Scientific, catalog no. A-31571, 1:1000 dilution]. After incubation, cells were washed six times for 5 min with 1× PBS, 0.1% Triton X-100 and stained with DAPI (1 ng/μl) in 1× PBS for 8 min. After two washes with 1× PBS, coverslips were mounted on glass slides using ProLong Diamond Antifade Reagent (Thermo Fisher Scientific).

### smiFISH combined with IF

For smiFISH/IF experiments, the smiFISH technique was performed first, using the protocol described in the smiFISH paragraph with the following modification: After washing twice in prewarmed 1× SSC, 15% formamide solution, cells were washed twice in 1× PBS before proceeding with the IF protocol described in the previous paragraph.

### smiFISH/IF image acquisition by confocal microscopy

Images were acquired using a confocal laser scanning Leica TCS SP8 inverted microscope (Leica Camera AG) with a plan apochromatic 63×/1.40 oil immersion objective and 2× zoom. Normal photomultipliers (PMTs) were used to detect telomeres and DAPI signals, while a hybrid detector (HyD) was used to image TERRA and hTR smiFISH signals. Optimal acquisition parameters including nanometer range of excitation, gain, offset, and laser power were optimized for the visualization of each fluorophore at the beginning of the acquisition and maintained throughout each experiment. Z stack images were acquired in each experiment with a 0.3-μm step and a 2048 × 2048 pixel size resolution using Leica Application Suite X (LAS X) imaging software.

Image postprocessing, including background subtraction, was performed by Fiji software (version 2.3.0/1.53o). For the quantification of hTR and TERRA single-molecule signals, analysis of the image stacks in 3D was performed using the 3D ImageJ Suite plugin (*74*). Two semiautomated ImageJ macros were used to analyze images. For colocalization analyses, an initial segmentation of fluorescent signals was performed followed by evaluation of their spatial distribution through distance analysis algorithm using DiAna plugin (*75*). Briefly, after determining the optimized threshold and background subtraction for all the signals, DiAna segmented and labeled each object (signal) of two different images at a time and evaluated the overlaps between them. The threshold and size parameters used for the object segmentation were kept constant for all the images. Two objects (A and B) are considered overlapping by DiAna when at least one voxel of object A colocalizes with one voxel of object B. DiAna output reports the number of colocalization events, their *x*,*y*,*z* position, and signal-specific integrated density. To evaluate the statistical significance of colocalizations between experimentally obtained image A and image B, this tool first computes distance analyses between objects in image A and image B, verifying the presence of colocalization events. Then, a shuffle function is applied to image B to generate 100 different images “B-shuffled,” in which the objects of image B are randomly redistributed. At this point, the software evaluates distances between objects in experimental image A and in all the 100 images B-shuffled. Then, it represents the cumulative distribution of these distances as a mean flanked by 95% confidence interval. Considering as null hypothesis that the experimental data are due to randomness, if the distribution of the distances from experimental images is outside the confidence interval obtained for shuffled images (in which object locations are random and thus colocalization is due to chance), there is less than 5% chance (*P* < 0.05) that the observed distribution is random (*75*).

### smiFISH/IF image acquisition by 3D-SIM

smiFISH/IF experiments were performed as described above in the smiFISH/IF paragraph on cells plated on 18-mm high-precision 1.5H glass slides (Marienfeld). Images were acquired in 3D-SIM mode using a Nikon N-SIM S microscope system equipped with a CFI Apo TIRF 100XC Oil (NA 1.49) objective and a Hamamatsu sCMOS ORCA-Flash 4.0 V2 digital camera. Images were acquired in z stacks with 0.1-μm step. Acquisition settings for each channel were optimized at the beginning and maintained for each experiment. Reconstruction was performed with the stack reconstruction method of the Nikon NIS-Elements AR software (version 5.41.02). Auto setting was selected for the illumination modulation contrast (IMC), while the high-resolution noise suppression (HRNS) was set to 1.

Images were analyzed using Fiji software. Two semiautomated ImageJ macros were used to analyze images. First, RAP1 and TERRA foci were segmented choosing appropriate thresholds for each channel that have been maintained throughout the experiments. Then, colocalization analyses were performed using the DiAna plugin of ImageJ, as described in the previous paragraph. The presence of telomeric singlets or doublets was assessed by examining orthogonal views of each RAP1 foci. A doublet was scored every time two partially overlapping signals were recognized. Images were rendered in 3D using Volume View with alpha blending option from Nikon NIS-Elements AR software (version 5.41.02).

### DNA FISH combined with IF

DNA FISH/IF protocol was adapted from Rossiello *et al.* (*76*). Cells seeded on 22 × 22 mm glass coverslips were fixed in 1× PBS, 4% PFA solution for 20 min at room temperature and permeabilized in 1× PBS, 0.2% Triton X-100 solution for 10 min. Cells were blocked by incubation in 1× PBG buffer (0.2% fish gelatin, 0.5% BSA, 1× PBS) for 1 to 6 hours and incubated for 1 hour with anti-RAP1 primary antibody (rabbit anti–TERF2-IP antibody, from Novus Biological, catalog no. NB100-292; RRID: AB_10000825) diluted 1:500 in 1× PBG. Cells were then washed three times with 1× PBG and incubated for 1 hour with the secondary antibody (anti-rabbit AF647, from Thermo Fisher Scientific #A21245) diluted 1:1500 in 1× PBG. After washing two times in 1× PBG buffer and two times in 1× PBS, cells were postfixed and permeabilized simultaneously in 4% PFA, 0.1% Triton X-100 solution for 10 min at room temperature. At this point, samples were incubated for 30 min at room temperature in 10 mM glycine solution diluted in milliQ water and washed 3 times for 5 min in 1× PBS. Coverslips were then inverted cell-side down on a 40-μl drop of hybridization mixture [70% formamide, 1× blocking reagent (Roche), 10 mM tris-HCl (pH 7.4), 0.5 μM telomeric PNA FAM probe] spotted on a precleaned glass slide, placed on a heat block for 5 min at 80°C, and incubated in a humidified chamber for 2 hours at room temperature protected from light. Following hybridization, coverslips were placed in a six-well plate and washed two times for 15 min with Wash Solution I [70% formamide, 0.1% BSA, 10 mM tris-HCl (pH 7.4)] and three times with Wash Solution II [100 mM tris-HCl (pH 7.4), 150 mM NaCl, 0.07% Tween 20]. After a quick rinse with 1× PBS, cells were stained for 8 min with DAPI (1 ng/μl) in 1× PBS, washed again with 1× PBS, and finally mounted on precleaned glass slides using ProLong Diamond Antifade Mountant. All images were acquired as a z stack on a spinning disk Eclipse Ti2 inverted microscope (Nikon Instruments Inc), equipped with Lumencor Spectra X Illuminator as light-emitting diode (LED) light source, an X-Light V2 Confocal Imager, and an Andor Zyla 4.2 PLUS sCMOS monochromatic camera using a Plan Apochromatic 100×/1.45 oil immersion objective. Images were analyzed using Fiji software.

### dCas9 sgRNA design

Oligonucleotides containing 20-mer guide sequence targeting a region of subtelomere 15q repeated 71 times at ~220-kb distance from the telomere were cloned in pUC57-attbU6-sgRNA-BbsI plasmid (gift of E. Bertrand) using the Bbs I restriction site. sgRNA sequence is shown in table S3.

### Live-cell imaging and TERRA MCP-sfGFP particle tracking

TERRA-MS2 cells previously generated in the AGS cell line (*61*, *62*) were transduced with MCP-sfGFP lentiviral vector particles. Cell transduction was performed in complete medium containing polybrene (8 μg/ml). Twenty-four hours after transduction, the viral vector particle–containing medium was replaced with fresh complete medium. Forty-eight hours after transduction, cells were sorted by fluorescence-activated cell sorting (FACS) to collect GFP-positive cells, which were cultured in complete medium for an additional 7 days. Cells were then transduced with TRF1-mCherry lentiviral vector particles and cotransfected with plasmids expressing dCas9-BFP and a sgRNA targeting subtelomere 15q.

For live-cell imaging, cells were plated in a 35-mm-diameter glass bottom dish (Ibidi GmbH 2020001). Acquisition parameters, exposure time, and light source intensity were optimized at the beginning of the acquisition to implement the visualization of each fluorophore and then maintained throughout the experiment. Live-cell imaging was performed using a Zeiss Axio-Observer Z1 microscope with a spinning disk connected to a 16-bit Evolve Electron Multiplying CCD Camera (EMCCD) camera. Images were acquired with alpha Plan Apochromat 100×/1.46 oil objective in spinning disk modality with a Yokogawa CSU-X1 rotating disc with 50-μm-diameter apertures (pinholes). A black incubator cabinet was used to keep cells at 37°C. Before imaging, cell culture medium was changed for transparent (without phenol red) medium with the addition of 5% Hepes 500. Live-cell imaging of TERRA molecules in TERRA-MS2 cells expressing MCP-sfGFP, TRF1-mCherry, dCas9-BFP, and subtelomere 15 sgRNA, was performed using a single camera mode, 10 frames (56.34 s), 10 slices (7.65 μm), laser light source intensity 70.3%, exposure time 150 ms, EM Gain 200, and binning mode 1.1. A z stack of subtelomere 15q (dCas9-BFP) and telomeric (TRF1-mCherry) signals was taken at the beginning and at the end of the TERRA-TRF1-mCherry imaging to identify colocalization or proximity of TERRA particles with subtelomere 15q and casual movement of the particles themselves. TERRA particles were considered in proximity when localized at 0.6-μm distance from the telomere 15q signal.

Image analysis was performed using Fiji software. The colocalization analysis of TERRA and telomeres in cotransfected cells was performed through a merge of the same z plane from the channels of interest. Validation of CRISPR-dCas9 system using pHR-SFFV-dCas9-BFP plasmid and 15q subtelomeric-targeting sgRNA in TERRA-MS2 cells was performed verifying the overlapping signals from TRF1-mCherry (telomeres) and dCas9-BFP (subtelomere 15q).

For TERRA particles analysis, first we verified the overlapping signal among TRF1-mCherry and dCas9-BFP. Then, a region of interest (ROI) representing the subtelomere 15q and telomeric signals was drawn manually or through the use of TrackMate. ROIs for all TERRA particles were designed manually, moving through every time point for each z plan, or through the use of TrackMate. The final analysis was performed visualizing all the ROIs at the same time. Overlapping TERRA ROIs were considered as a single TERRA molecule. A necessary condition to consider TERRA ROIs and subtelomere 15q ROI “in proximity” or “overlapping” was their simultaneous presence in the same z plan.

### Live-cell imaging and hTR MCP-sfGFP particle tracking

HeLa hTR^5’MS2^ + hTERT cells, described in (*37*), stably express MCP-sfGFP to label hTR, TRF1-mCherry to detect telomere foci, and CDT1-mCherry to mark cells in S-phase or G_2_. HeLa hTR^5’MS2^ + hTERT cells plated on glass-bottom 35 mm dishes (Fluorodish FD35-100, World Precision Instruments) in growth medium were transfected with TERRA-ASO, ASO SCR or with Oligofectamine alone (UNT) for 48 hours, as described in the dedicated paragraph. To image the cells, 25 mM Hepes (pH 7.4) was added to the growth medium, and the 35-mm dishes were placed in a 37°C heated chamber on a Zeiss Axio-Observer Z1 Yokogawa CSU-X1 spinning disk confocal microscope with EMCCD Evolve camera (Photometrics, 512 × 512 pixels, 16 μm) with a 100× 1.46–numerical aperture (NA) objective, 0.133-μm pixel size, using the 488-nm 100-mW and 561-nm 40-mW diode lasers. Focus was maintained with the Zeiss Definite Focus LED IR 835-nm laser. Images were acquired every 5 s for a total duration of 5 min. The hTR-telomere movies were corrected for acquisition photobleaching and analyzed with ImageJ TrackMate and CoPixie colocalization software. GraphPad Prism was used for survival probability analysis (1-CDF) of hTR dwell time at telomeres. Curve fitting was performed with an equation for two-phase exponential decay using least squares regression and weighting by 1/*Y*^2^ for optimal curve fitting (*R*^2^ > 0.99). The following constrains were imposed on the parameters: Plateau was fixed at 0 and KSlow > 0. The residence time (τ_fast_ and τ_slow_) and half-life (fast and slow) were derived from the model. For the cumulative dwell-time analysis, data were fitted to a Kaplan-Meier survival curve.

### Genomic DNA extraction

Genomic DNA was isolated from cells using phenol/chloroform/isoamyl alcohol (25:24:1, Fisher Scientific) according to the manufacturer’s instructions. Briefly, cells were collected by scraping in ice-cold 1× PBS and the pellet resulting from centrifugation was resuspended in Lysis Buffer [10 mM tris-HCl (pH 7.4), 10 mM EDTA, 0.5% SDS, 10 mM NaCl, 1:750 RNase A (20 mg/ml)] and incubated at 37°C. After 1 hour, 0.2% proteinase K (Invitrogen) was added to lysates, mixed by inversion, placed at 50°C for 3 hours, and incubated overnight at 37°C. The following day, the samples were heated at 50°C for 3 hours. At this point, phenol/chloroform/isoamyl alcohol (Fisher Scientific) was added in a ratio 1:1 to the samples. The tubes were shaken vigorously and centrifuged at 15,000*g* for 15 min at room temperature. The upper aqueous phase was collected in a new tube, and phenol/chloroform/isoamyl alcohol extraction was repeated. After collecting the aqueous upper phase into a new tube, chloroform was added in a ratio 1:1 to the sample. Samples were vortexed and centrifuged, and the resulting upper phase was harvested and precipitated at −20°C overnight using 1:10 of total volume of 3 M NaAc (pH 5.2) and two volumes of 100% ethanol. After one wash step with 70% ethanol, pellets were resuspended in the appropriate volume of nuclease-free water. Concentration and purity were assessed using NanoDrop 1000 spectrophotometer (Thermo Fisher Scientific), and DNA integrity was verified by electrophoresis on a 1% agarose 0.5× Tris/Boric Acid/EDTA (TBE) gel.

### TRF analysis by Southern blotting

Genomic DNA (10 μg) digested with Rsa I (Thermo Fisher Scientific) and Hinf I (NEB) restriction enzymes overnight at 37°C were run on a 0.8% agarose gel in 0.5× TBE buffer at 25 V for 18 hours. The day after, the gel was stained in Atlas ClearSight dye (25 μl/100 ml) dissolved in 0.5× TBE for 30 min, quickly rinsed with 0.5× TBE and deionized water, and subsequently denatured in denaturing solution (0.5 M NaOH,1.5 M NaCl) for 1 hour at room temperature. After a wash step with deionized water, the gel was soaked two times for 30 min in neutralizing solution [0.5 M tris-HCl (pH 7.5), 1.5 M NaCl] on a rocking platform and equilibrated for 10 min in 10× SSC. The DNA was blotted on an Amersham Hybond H+ membrane (GE Healthcare) overnight at room temperature through capillarity. The following day, DNA was cross-linked to the membrane with ultraviolet (UV) light (365 nm) for 1 min at 120 mJ/cm^2^ on the UVP CL-1000L Longwave (Fisher Scientific), prehybridized for at least 1 hour at 50°C with preheated hybridization buffer (5× SSC, 0.1% sarcosyl, 0.04% SDS), and hybridized overnight at 50°C with Telo DIG-NICK probe (40 ng/ml) previously denatured at 95°C for 5 min and then diluted in hybridization buffer. Probe preparation is described in the next paragraph. The next day, the membrane was washed with 2× SSC, 0.1% SDS for 5 and 15 min at room temperature, and twice with 0.5× SSC, 0.1% SDS for 5 min. Subsequently, two washes of 15 min at 42°C with preheated 0.5× SSC, 0.1% SDS were performed. After rinsing it quickly in 2× SSC, the membrane was blocked for 1 hour in blocking solution [1% blocking reagent, Roche, in maleic acid buffer (100 mM maleic acid, 250 mM NaCl, pH 7.5)] and incubated with anti–digoxigenin-alkaline phosphatase (anti–DIG-AP) antibody (Roche) (1:10,000 dilution) diluted in blocking solution for at least 2 hours. Before detecting the chemiluminescent signal, the membrane was washed two times with 0.1 M maleic acid, 0.15 M NaCl (pH 7.5), 0.3% Tween 20, equilibrated for 2 min in AP buffer [0.1 M tris-HCl, 0.1 M NaCl (pH 9.5)], and incubated with CDP-Star Chemiluminescence Substrate (Roche) for 5 min. The membrane was exposed to UV light for 2 hours using a ChemiDoc XRS^+^ (Bio-Rad).

The average length of telomeres was assessed by using Image Lab 6.1 software (Bio-Rad). The image was divided into lanes (one for each sample) using the “Lanes and bands” tool. Subsequently, for each sample both the start and the end of the corresponding telomeric smear were defined using the “Lanes and bands” command which resulted in a table reporting the values of relative front detected for each smear. The relative front parameter indicates the relative movement of the analyzed band from the top to the bottom. The correlation between relative front value and kilobase was calculated by assessing the molecular weight marker.

### TRF probe preparation

The telomeric probe was generated starting from the psXNeo-1.6-T2AG3 plasmid and labeled through DIG-Nick Translation Mix for in situ probes (Roche) according to the manufacturer’s instructions. Briefly, psXNeo-1.6-T2AG3 plasmid (Addgene #12403) was digested using Bgl I and Sma I restriction enzymes (NEB). The band corresponding to the 1.6-kb T2AG3 insert was extracted from the gel using Wizard SV Gel and PCR Clean-Up System (Promega). Extracted DNA (1 μg) was mixed with 4 μl of DIG-Nick Translation Mix (Roche) and incubated at 15°C for 2 hours. The reaction was then chilled in ice and stopped by boiling it at 65°C for 10 min after the addition of 1 μl of 0.5 M EDTA (pH 8).

### Cell cycle analyses by flow cytometry

Cells were harvested by trypsinization, washed with 1× PBS, and fixed with ice-cold 70% ethanol in 1× PBS for at least 20 min at −20°C. Fixed cells were centrifuged for 3 min at 1000*g*, washed two times with 1× PBS, and stained with propidium iodide (10 μg/ml) (Invitrogen) for 30 min at 37°C in the presence of PureLink RNase A (100 μg/ml) (Invitrogen). Cells were analyzed in a flow cytometer (FACSCanto, BD Bioscience). Data analysis and figure preparation were performed using FlowJo software (FlowJo LLC).

### TERRA knockdown by ASO

For TERRA depletion, cells were grown to 50% confluence on six-well plates and transfected with 200 nM ASO LNA Gapmers (Qiagen) specifically designed to target TERRA (*77*) using Oligofectamine transfection reagent (Thermo Fisher Scientific) following the manufacturer’s instructions. A Scrambled LNA was used as negative control (Qiagen). RNA extraction or cell fixation was performed 24 or 48 hours after transfection. Sequences of oligos are reported in table S3.

### RNA isolation

Total RNA was isolated from cells using TRIzol reagent (Thermo Fisher Scientific) according to the manufacturer’s instructions. Briefly, cells cultured on 10 cm petri dishes were lysed with 1 ml of TRIzol Reagent, scraped, and collected in sterile tubes. After 10 min incubation at room temperature, 200 μl of chloroform was added to the lysates, and the samples were mixed by vortexing and incubated for 15 min at room temperature. After centrifugation at 4°C, the upper aqueous phase was collected in a new tube, and 500 μl of isopropanol was added to the tube and mixed by inversion. After a 10-min incubation, samples were centrifuged at 4°C. The pellet was washed two times with 70% ethanol and resuspended in the appropriate volume of nuclease-free water. RNA concentration and purity were assessed using NanoDrop 1000 spectrophotometer. RNA integrity was confirmed by electrophoresis on a 1% agarose 1× Mops pH 7 (20 mM Mops, 2 mM sodium acetate, 1 mM EDTA) gel.

### Reverse transcription quantitative polymerase chain reaction

Total RNA was treated with deoxyribonuclease (DNase) I (Thermo Fisher Scientific) for 1 hour at 37°C. The integrity of DNase I–treated RNAs was confirmed by running on a 1% agarose 1× Mops gel. RNAs were reverse-transcribed with hTR RT primer, TERRA RT primer, or random hexamers using the SuperScript III RT enzyme (Thermo Fisher Scientific), following the manufacturer’s protocol. hTR and TERRA expression levels were monitored via quantitative PCR using KAPA SYBR FAST qPCR Mastermix (KAPA Biosystems) by running 50 ng of RT reaction in triplicates in a CFX Touch Real-Time PCR Detection System (Bio-Rad) or a QuantStudio5 PCR machine (Thermo Fisher Scientific). Ct values were extracted using a Bio-Rad CFX Manager Software or QuantStudio Design and Analysis (Thermo Fisher Scientific), expression values were normalized to the geometric mean of U6 housekeeping gene, and the relative quantification was presented as linearized Ct values (2^−ΔΔCt^), normalized to the control sample. Primers used in this study are listed in table S3.

### Northern blotting

Total RNA was extracted from cells using TRIzol reagent as described in the “RNA isolation” section. Total RNA (20 μg) was treated with 2.5 μl of DNase I enzyme for 1 hour at 37°C with addition of 0.5 μl of RiboLock Rnase inhibitor (Thermo Fisher Scientific) to the reaction. DNase I–treated RNA integrity was confirmed by electrophoresis on a 1% agarose 1× Mops gel. As a negative control, 20 μg of the same RNA was incubated with PureLink Rnase A (2 mg/ml) (Invitrogen) for 1 hour at 37°C and subsequently treated with 2.5 μl of DNase I enzyme. RNAs were denatured for 5 min at 65°C after adding in 2× RNA Loading Dye and subsequently loaded on a 1.2% agarose 1× Mops gel containing 2% formaldehyde and the Atlas ClearSight (Bioatlas) intercalating dye. After running, the RNA was blotted on an Amersham Hybond H+ membrane (GE Healthcare) by capillarity overnight using 10× SSC buffer (3 M NaCl, 0.3 M Sodium Citrate). Then, RNAs were cross-linked to the membrane with UV light (365 nm) for 1 min at 120 mJ/cm^2^ on the UVP CL-1000L Longwave (Fisher Scientific), prehybridized for at least 1 hour at 42°C with preheated Church hybridization buffer (1% BSA, 1 mM EDTA, 0.5 M phosphate buffer, 7% SDS), and hybridized overnight at 45°C with a TERRA-targeting probe labeled with radioactive ^32^P (TERRA probe sequence: CCCTAACCCTAACCCTAACCCTAACCCTAA). The following day, the membrane was washed twice with 2× SSC, 0.1% SDS for 5 and 15 min at room temperature, followed by two washes with 0.5× SSC, 0.1% SDS for 5 min and two washes with preheated 0.5× SSC, 0.1% SDS for 15 min at 42°C. After rinsing it quickly in 2× SSC, the membrane was wrapped in saran wrap and maintained in an exposure cassette for up to 4 days. Detection of the radioactive signal was performed using a Typhoon.

### Statistical analysis

GraphPad Prism 9 (license: GPS-1810727-TFRO-F3E3C) was used to produce graphs and statistical analyses. Normality of datasets was determined by Shapiro-Wilk test. Details about the statistical test performed for each experiment are provided in the figure legends. *P* values are indicated as follows: *P < 0.05, **P < 0.01, ***P < 0.001, ****P < 0.0001, ns: not significant. Significance of colocalization events detected by smiFISH and smiFISH/IF experiments was confirmed running the DiAna plugin of ImageJ.

## Supplementary Materials

### This PDF file includes:

Figs. S1 to S7 Tables S1 to S3 Legends for movies S1 to S6 References
