# Supplementary material for: TERRA transcripts localize at long telomeres to regulate telomerase access to chromosome ends
Source: Sci Adv. 2024 Jun 12;10(24):eadk4387. doi: 10.1126/sciadv.adk4387 (PMC11168465; doi:10.1126/sciadv.adk4387)
Supplement: Supplementary file 1 — Figs. S1 to S7 Tables S1 to S3 Legends for movies S1 to S6 References [file sciadv.adk4387_sm.pdf]

Science Advances
AAAS

Supplementary Materials for

TERRA transcripts localize at long telomeres to regulate telomerase access to chromosome ends

Nicole Bettin *et al.*

Corresponding author: Emilio Cusanelli, emilio.cusanelli@unitn.it

Sci. Adv. **10**, eadk4387 (2024)
DOI: 10.1126/sciadv.adk4387

The PDF file includes:

Figs. S1 to S7
Tables S1 to S3
Legends for movies S1 to S6
References

Other Supplementary Material for this manuscript includes the following:

Movies S1 to S6

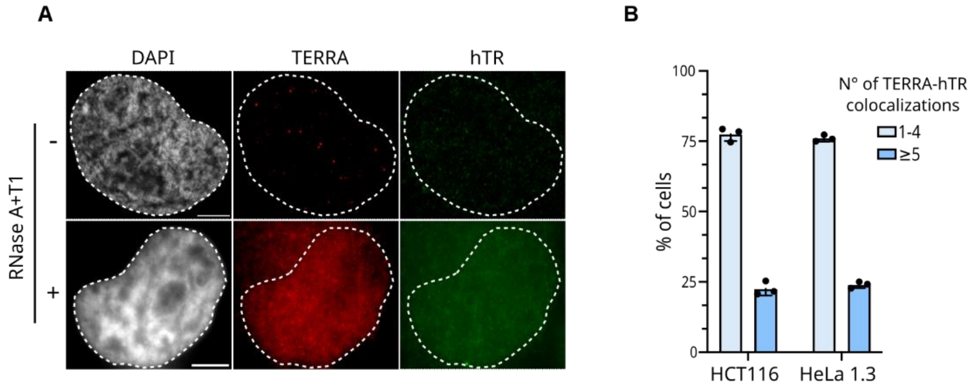

Fig. S1. Detection of TERRA and hTR foci by smiFISH. A) Treatment with RNase A and T1 was performed in HeLa cells to confirm the specificity of the signals. Scale bar: 5 µm. **B**) Quantification of the number of TERRA-hTR colocalizations per cell in HCT116 and HeLa 1.3 cell lines. Data shown represents mean ± SD from 3 biological replicates for each cell line, corresponding to a total of 227 HCT116 cells and 144 HeLa 1.3 cells analyzed.

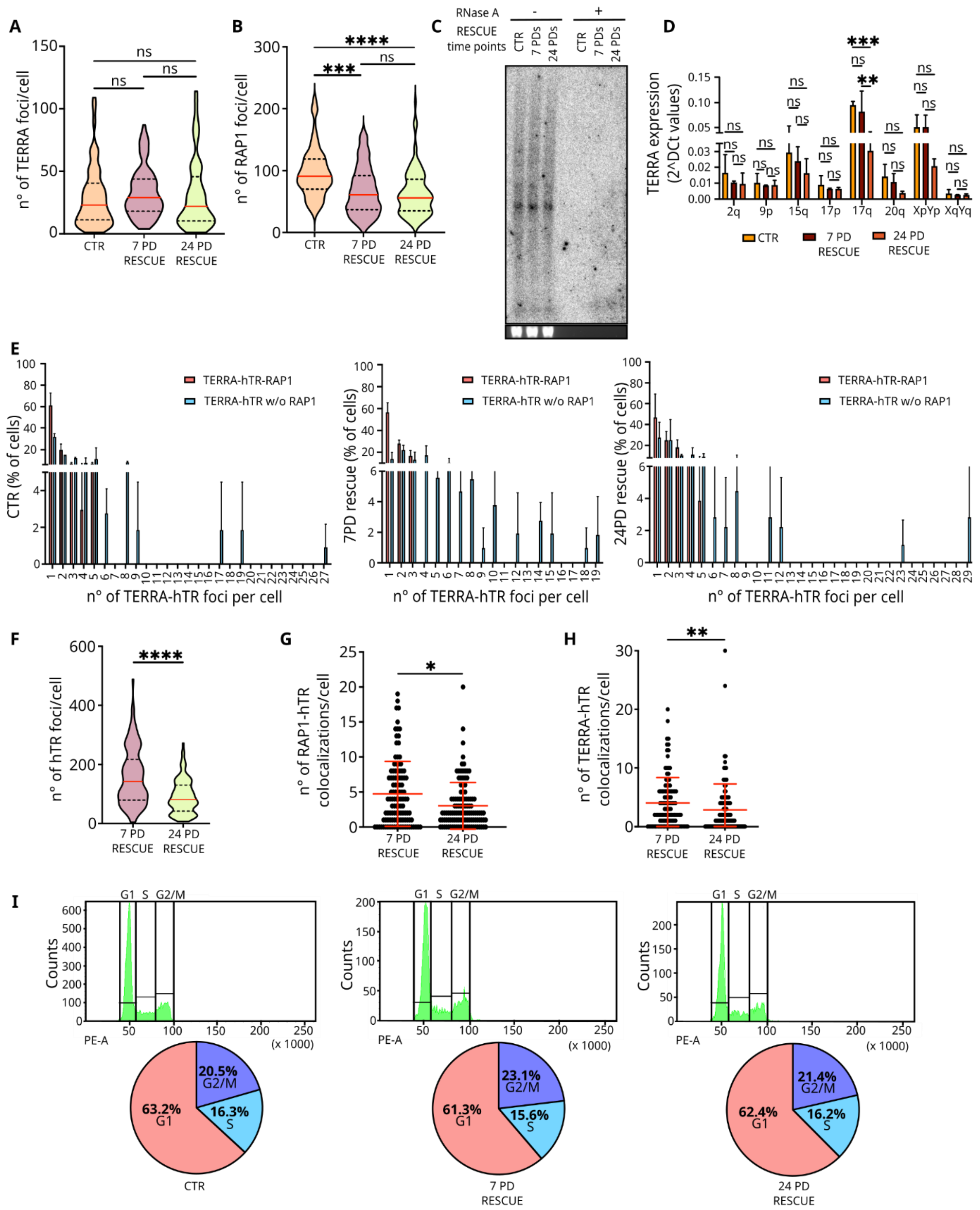

Fig. S2. smiFISH/IF, RT-qPCR and cell cycle analyses of BIBR-Rescue experiment. A-B) Quantification of TERRA (A) and RAP1 (B) foci per nucleus detected by smiFISH/IF in the indicated samples. Mean ± SD is shown from 2 independent biological replicates for a total of 124 CTR cells, 111 7PD rescue cells, and 112 24PD rescue cells. An unpaired non-parametric Kruskal-

Wallis test coupled with a post hoc Dunn's multiple comparison test was used for statistical significance. p-value= ns: not significant, *** <0.001, **** <0.0001. **C**) Northern blot analysis of TERRA levels in the indicated samples. Bottom image shows 18S rRNA band upon gel run. RNase A treatment was performed to confirm specificity of the signal. **D**) RT-qPCR analysis of TERRA expression from the indicated telomeres in CTR, 7PD rescue and 24PD rescue cells. Mean ± SD from two biological replicates. Unpaired t-test p-values= ** <0.01, *** <0.001, ns: not significant. **E)** Quantification of the number of TERRA-hTR foci per nucleus detected at telomeres and outside telomeres. Mean ± SD; n= 2; 124 CTR cells, 111 7PDs rescue cells and 112 24PD rescue cells were analyzed. **F-H**) Quantification of the number of hTR (F), telomeric hTR (G) and TERRA-hTR (H) foci during telomere re-lengthening in 7PD and 24PD rescue cells. Mean ± SD; n= 2; 124 CTR cells, 111 7PDs rescue cells and 112 24PD rescue cells were analyzed. Mann Whitney test p-values= *: p< 0.05, **: p<0.01, ****: p<0.0001. **I)** Cell cycle profile analyses by FACS of CTR, 7 PD rescue and 24 PD rescue cells. The percentage of cells in G1, S and G2/M phases is shown for each condition.

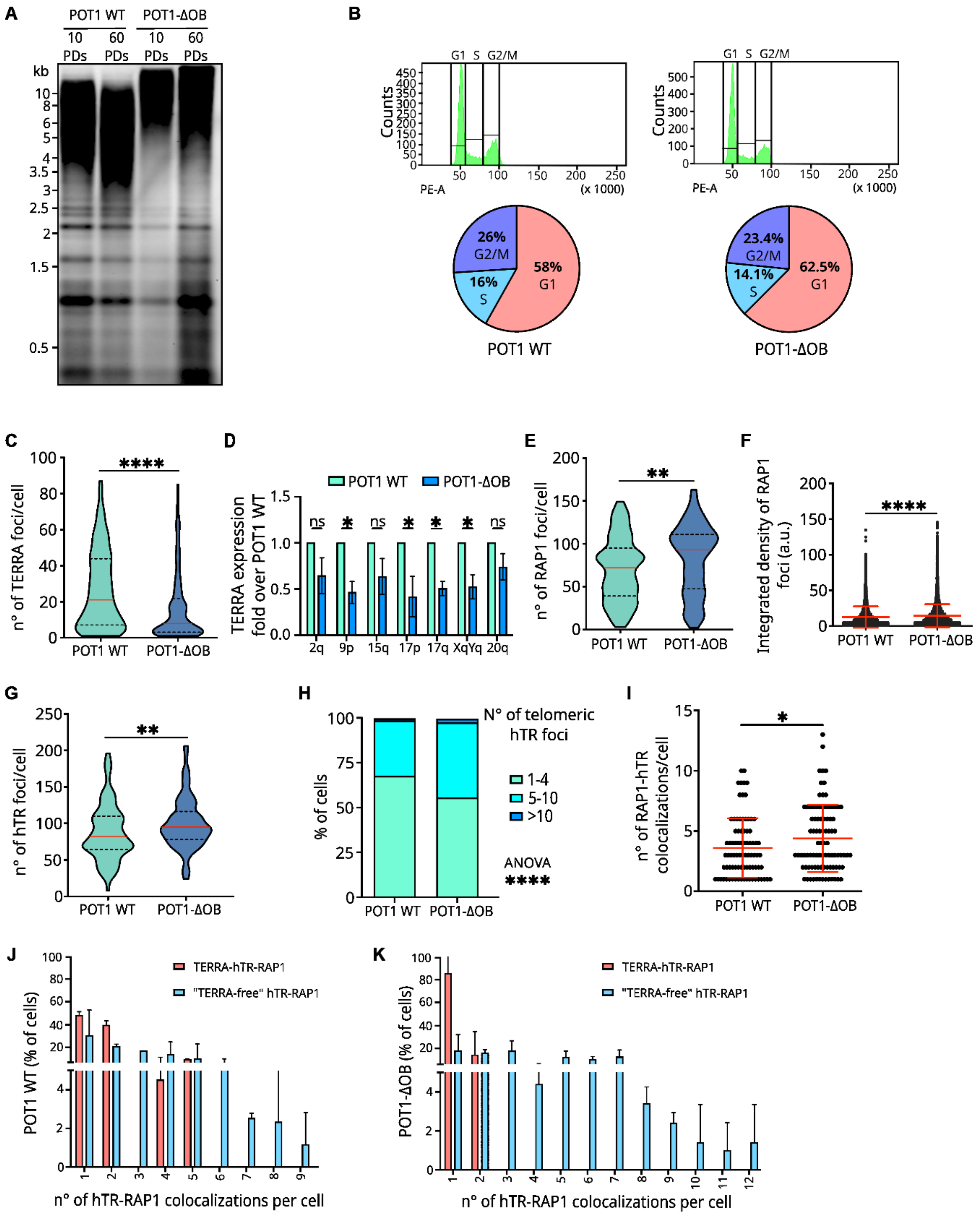

Fig. S3. Validation experiments of POT1 WT and POT1-ΔOB cells. A) Measurement of telomere length by Terminal Restriction Fragment (TRF) analysis through Southern blot in POT1 WT and POT1-ΔOB cells cultured for 10 and 60 population doublings (PDs). **B**) Cell cycle profile

analyses by FACS of POT1 WT and POT-ΔOB expressing cells. The percentage of cells in G1, S and G2/M phases is shown. **C)** Quantification of the number of TERRA foci detected per nucleus in POT1 WT and POT1-ΔOB cells by smiFISH/IF. Mean ± SD is shown from 3 independent biological replicates for a total of 151 POT1 WT cells and 148 POT1-ΔOB cells analyzed. Mann Whitney test p-value= ****: < 0.0001. **D)** RT-qPCR analyses of TERRA expression from the indicated telomeres in POT1 WT and POT1-ΔOB cells. Mean ± SD were calculated from three independent experiments. Technical triplicates were analyzed in each experiment. Unpaired t-test was used for statistical analyses: p-value= *: <0.05, ns: not significant. **E-F**) Quantification of the number (E) and integrated density (F) of RAP1 foci detected per nucleus in POT1 WT and POT-ΔOB cells by smiFISH/IF. Mean ± SD is shown from 3 independent biological replicates for a total of 151 POT1 WT cells and 148 POT1-ΔOB cells analyzed. Mann Whitney test p-values= **: <0.01, ****: < 0.0001. **G)** Quantification of the number of hTR foci detected per nucleus in POT1 WT and POT-ΔOB cells by smiFISH/IF. Mean ± SD is shown from 2 independent biological replicates for a total of 100 POT1 WT cells and 105 POT1 ΔOB cells analyzed. Mann Whitney test p-value= **: <0.01. **H**) Distribution analysis of the number of telomeric hTR foci in POT1 WT and POT-ΔOB cells represented as percentage of cells showing 1-4, 5-10 or >10 hTR foci at telomeres. Two-way ANOVA test p-value= ****: <0.0001. **I)** Quantification of the number of telomeric hTR foci per cell in POT1 WT and POT-ΔOB cells. A total of 100 POT1 WT cells and 105 POT1-ΔOB cells were analyzed in 2 biological replicates. Unpaired non-parametric Kruskal-Wallis test p-value *= ≤0.05. **J-K)** Number of hTR-RAP1 colocalizing foci with and w/o TERRA in POT1 WT (J) and POT1-ΔOB (K) cells, related to Figure 3H.

A

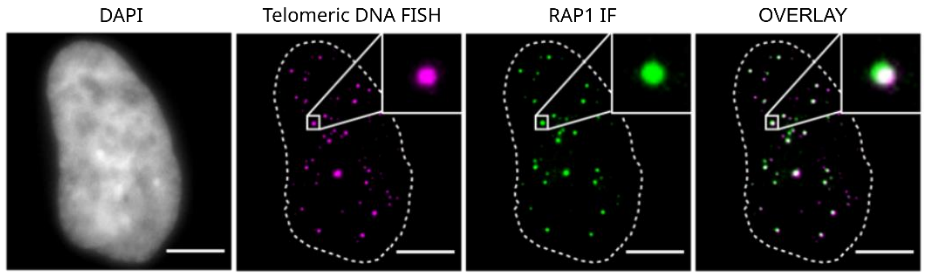

B

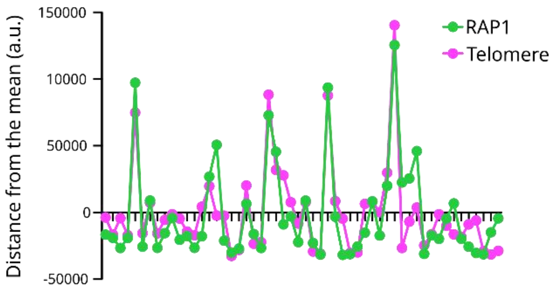

C

RAP1 IF signals vs telomeric DNA-FISH signals	
Spearman r	0.5793
95% C.I.	0.5587 to 0.5992
p-value	< 0.0001
XY pairs	4378

D

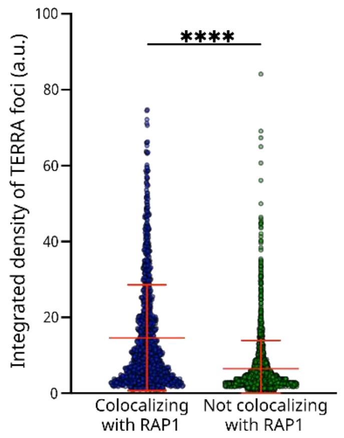

E

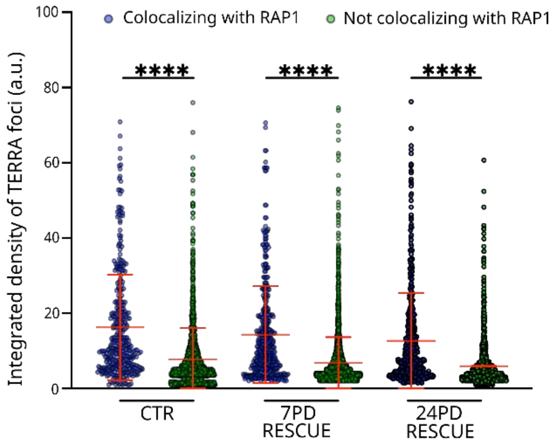

Fig. S4. Detection of telomeres by telomeric DNA FISH and RAP1 IF (DNA FISH/IF) in HeLa cells. A) Representative image of DNA FISH/IF experiment. An example of a colocalization event between the telomeric DNA FISH signal and the IF signal is shown. DAPI is used to stain nuclei. Scale bar: 5 µm. **B**) Integrated density quantification of telomeric DNA FISH and RAP1 IF foci from DNA FISH/IF experiments in HeLa cells. Data is shown as distance of integrated density of each telomeric focus from the average telomere integrated density (set to zero) calculated by telomeric DNA FISH and RAP1 IF. Each dot represents a single telomere focus. RAP1 foci are displayed in green, and telomeric DNA FISH foci are shown in magenta. The

analysis of a representative cell is shown. **C**) Comparison of integrated density quantification analyses of telomeric DNA FISH and RAP1 IF from DNA FISH/IF experiments performed in HeLa cells, as in B. Spearman's rank correlation was calculated from the analyses of 134 nuclei, corresponding to a total of 6745 RAP1 foci and 13985 telomeric DNA FISH foci analyzed. Sperman's coefficient = 0.5793. **D-E**) Integrated density quantification of TERRA foci colocalizing and not colocalizing with RAP1 foci in HeLa cells (D) and in HeLa CTR, 7PD rescue and 24PD rescue cells (E). Each dot represents a single TERRA signal. Mean ± SD is shown from 2 biological replicates, corresponding to a total of 102 (HeLa), 124 (CTR), 111 (7PD rescue), and 112 (24PD rescue) cells analyzed. The Mann Whitney test was used to assess statistical significance. p-value= ****: <0.0001.

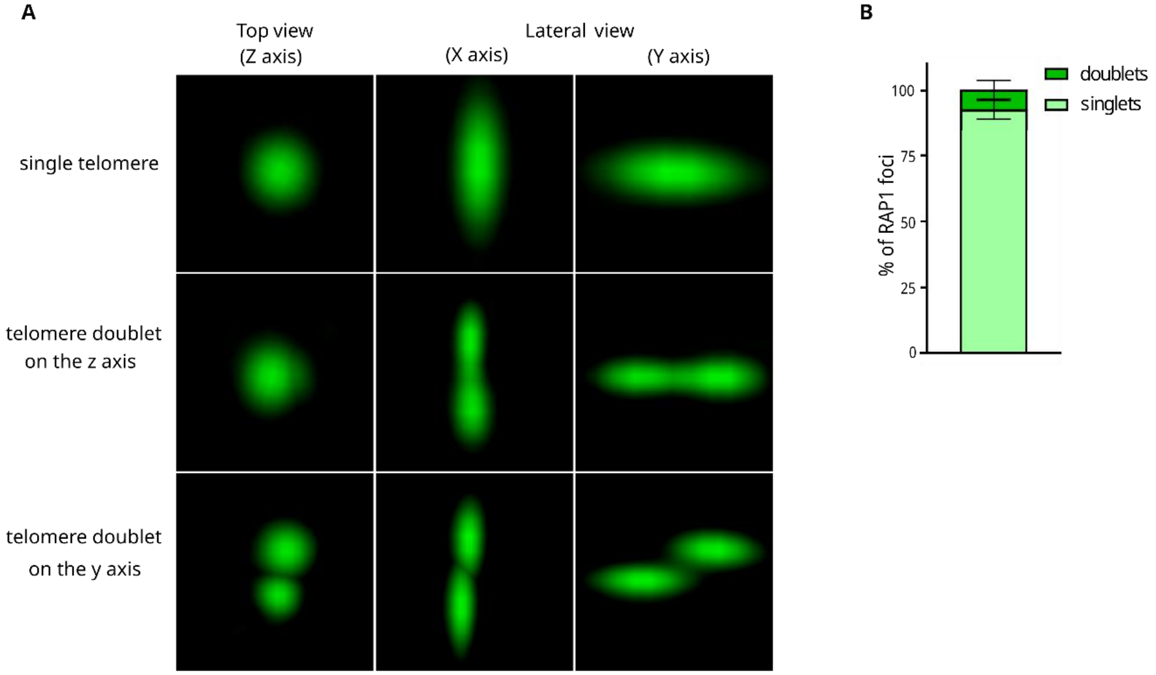

Fig. S5. Detection of telomere singlets and doublets by RAP1 IF experiments and 3D structured illumination microscopy (3D-SIM) in HeLa cells. A) Representative images of RAP1 IF detected by 3D-SIM. Two different telomere doublets, one on the z axis and a second on the y axis, and a telomere singlet are shown. View from z, x, and y axis are displayed. **B)** Quantification of the number of telomere singlets and doublets detected in RAP1 IF experiments by 3D-SIM microscopy in HeLa cells. Data is shown as a percentage over the total number of telomeric foci detected. 7 cells were analyzed for a total of 608 RAP1 foci imaged in 2 biological replicates.

A

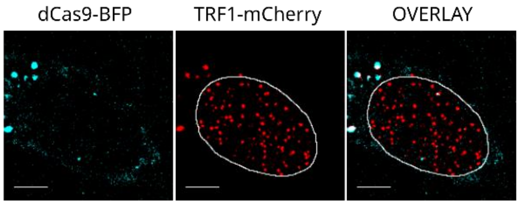

B

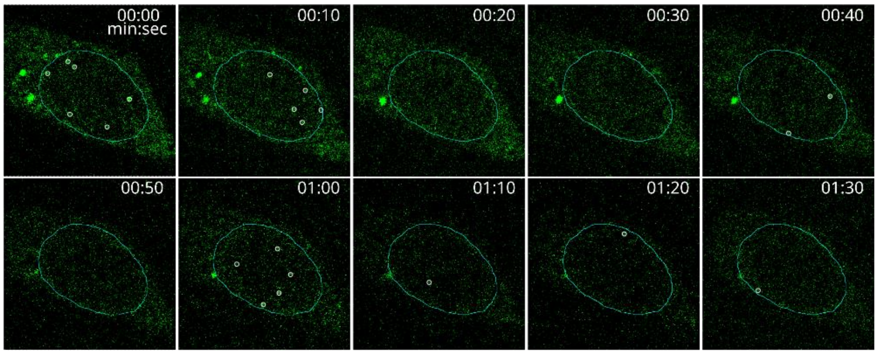

Fig. S6. Live-cell imaging of TERRA-MS2 cells. A) Still images of TERRA-MS2 cells expressing TRF1-mCherry, MCP-sfGFP and co-transfected with subtelomere 15q-targeting sgRNA and dCas9-BFP expressing vectors. Images show telomere foci visualized by TRF1-mCherry (red), subtelomere 15q foci detected by dCas9-BFP (blue) and the overlay. Maximum intensity projections of the z-stacks are shown. Image deconvolution was performed using AutoQuant software. Scale bar: 5 µm. **B)** Screenshots of Image J TrackMate spot detection used to identify TERRA MCP-sfGFP particles in timelapse image sets. The images correspond to the experiment shown in figure 6B. TERRA MCP-sfGFP particle detection and colocalization analysis with TRF1-mCherry spots was performed on raw images from the spinning disk confocal microscope.

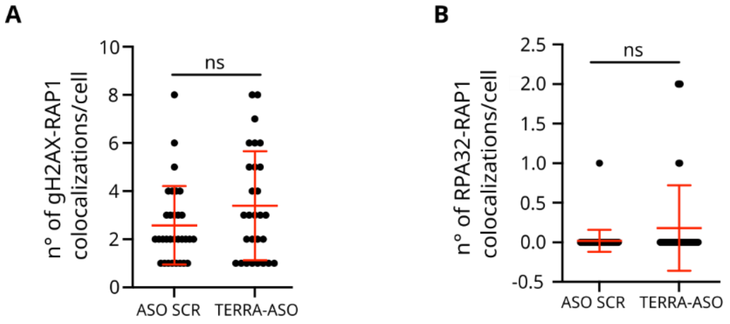

Fig. S7. TIF formation and RPA32 localization at telomeres in cells transfected with TERRA-ASO or ASO SCR. A) Quantification of the number of ɣH2AX foci colocalizing with telomeres in TERRA-ASO and ASO SCR cells. A total of 46 ASO SCR cells and 49 TERRA-ASO cells were analyzed. The Mann Whitney test was used to assess statistical significance. ns= not significant. **B)** Quantification of the number of RPA32 foci colocalizing with telomeres in TERRA-ASO and ASO SCR cells. A total of 52 ASO SCR cells and 44 TERRA-ASO cells were analyzed. The Mann Whitney test was used to assess statistical significance. ns= not significant.

Plasmid	Experiment	Source
pCMV-VSV-G	Expression of envelop protein for producing retroviral and lentiviral particles	Addgene #8454
psPAX2	Packaging plasmid for lentiviral vector production	Addgene #12260
pCMV-deltaR8.91	Lentiviral vector production	(*73*)
pLPC myc-hPOT1	Expression of POT1 WT	Addgene #12387
pLPC myc-hPOT1-ΔOB	Expression of POT1- ΔOB	Addgene #13241
pHR-SFFV-dCas9-BFP	Expression of dCas9-BFP	Addgene #46910
pUC57-attbU6-sgRNA-BbsI	Expression of subtelomere15q-targeting sgRNAs	kind gift of Edouard Bertrand
pHAGE2-EF1a-mCherry-hTRF1-IRES-blast	Expression of TRF1-mCherry	Chartrand lab
pHage-UBC-MCP-sfGFP	MCP-sfGFP expression	Chartrand lab

Table S1. Plasmids used in this study.

Probe name	Sequence (5'-3')
hTR Probe 1	GCATGTGTGAGCCGAGTCCTGGGTGCTTACACTCGGACCTCGTCGACATGCATT
hTR Probe 2	CGCGCGGGGACTCGCTCCGTTCCTTACACTCGGACCTCGTCGACATGCATT
hTR Probe 3	TTCCTGCGGCCTGAAAGGCCTGAACTTACACTCGGACCTCGTCGACATGCATT
hTR Probe 4	GGGCCAGCAGCTGACATTTTTTGTTTGCTTTACACTCGGACCTCGTCGACATGC ATT
hTR Probe 5	GGCTTTTCCGCCCGCTGAAAGTCAGCTTACACTCGGACCTCGTCGACATGCATT
hTR Probe 6	GTCCCACAGCTCAGGGAATCGCGCTTACACTCGGACCTCGTCGACATGCATT
hTR Probe 7	GCCCAACTCTTCGCGGTGGCAGTGTTACACTCGGACCTCGTCGACATGCATT
hTR Probe 8	GCGGCCTCCAGGCGGGGTTCGGGTTACACTCGGACCTCGTCGACATGCATT
hTR Probe 9	CCGCAGGTCCCCGGGAGGGGCGATTACACTCGGACCTCGTCGACATGCATT
hTR Probe 10	AGAATGAACGGTGGAAGGCGGCAGGCCTTACACTCGGACCTCGTCGACATGCA TT
hTR Probe 11	CGCCTAGCGCCTTCTCAGTTAGGGTTTTACACTCGGACCTCGTCGACATGCATT
hTR Probe 12	CCCCGAGAGACCCGCGGCTGACATTACACTCGGACCTCGTCGACATGCATT
hTR Probe 13	CCTCCGGAGAAGCCCCGGGCCGATTACACTCGGACCTCGTCGACATGCATT
hTR Probe 14	GAAAAACAGCGCGCGGGGAGCAAAAGCACTTACACTCGGACCTCGTCGACATG CATT
hTR Probe 15	ACAAAAAATGGCCACCACCCCTCCCTTACACTCGGACCTCGTCGACATGCATT
TERRA Probe	TAACCCTAACCCTAACCCTAACCCTAACCCTTACACTCGGACCTCGTCGACATG CATT
Secondary probe Y-AF647	/5AF647/AATGCATGTCGACGAGGTCCGAGTGTAA/3AF647Sp
Secodary probe Y-AF555	/5AF555/AATGCATGTCGACGAGGTCCGAGTGTAA/3AF555Sp

Table S2. List of primary and secondary probes used for smiFISH experiments.

Name	Sequence (5’-3’)	Experiment	Manufacturer	Reference
sgRNA_15q_fw	CACCGTCGGCAGAGC AGGCAGACCA	dCas9-BFP targeting to subtelomere 15q	Metabion	This study
sgRNA_15q_rev	AAACTGGTCTGCCTGC TCTGCCGAC	dCas9-BFP targeting to subtelomere 15q	Metabion	This study
TERRA 2q For	GCCTTGCCTTGGGAGA ATCT	qPCR	Metabion	*(81)*
TERRA 2q Rev	AAAGCGGGAAACGAA AAGC	qPCR	Metabion	*(81)*
TERRA 9p For	GAGATTCTCCCAAGGC AAGG	qPCR	Metabion	*(82)*
TERRA 9p Rev	ACATGAGGAATGTGG GTGTTAT	qPCR	Metabion	*(82)*
TERRA 15q For	CAGCGAGATTCTCCCA AGCTAAG	qPCR	Metabion	*(16)*
TERRA 15q Rev	AACCCTAACCACATG AGCAACG	qPCR	Metabion	*(16)*
TERRA 17p For	CTTATCCACTTCTGTC CCAAGG	qPCR	Metabion	*(82)*
TERRA 17p Rev	CCCAAAGTACACAAA GCAATCC	qPCR	Metabion	*(82)*
TERRA 17q For	GTCCATGCATTCTCCA TTGATAAG	qPCR	Metabion	*(81)*
TERRA 17q Rev	AGCTACCTCTCTCAAC ACCAAGAAG	qPCR	Metabion	*(81)*
TERRA 20q For	ACATGGGCGATACTC AGG	qPCR	Metabion	*(17)*
TERRA 20q Rev	CCCACTACTGTGCCTC AA	qPCR	Metabion	*(17)*
TERRA XpYp For	AAGAACGAAGCTTCC ACAGTAT	qPCR	Metabion	*(16)*
TERRA XpYp Rev	GGTGGGAGCAGATTA GAGAATAAA	qPCR	Metabion	*(16)*

TERRA XqYq For	GAAAGCAAAAGCCCCTCTGA	qPCR	Metabion	*(81)*
TERRA XqYq Rev	CCCCTTGCCTTGGGAGAA	qPCR	Metabion	*(81)*
TERRA RT	CCCTAACCCTAACCCTAACCCTAACCCTAA	TERRA reverse transcriptase	Metabion	*(63)*
hTR RT	CATGTGTGAGCCGAGTCCTG	hTR reverse transcription	Metabion	This study
hTR mature FOR	GCGAAGAGTTGGGCTCTGTCA	qPCR for hTR mature form	Metabion	*(78)*
hTR mature REV	TTCCTCTTCCTGCGGCCTGAAA	qPCR for hTR mature form	Metabion	*(78)*
hTR precursor FOR	GGGTGTGGGAGAACAGTCAT	qPCR for hTR precursor form	Metabion	*(79)*
hTR precursor REV	ACCTCTGGCATAAACCGATG	qPCR for hTR precursor form	Metabion	*(79)*
U6 FOR	GTGCTCGCTTCGGCAGCACA	qPCR for U6 (housekeeping control)	Metabion	*(80)*
U6 REV	GGAACGCTTCACGAATTTGCGTGTCAT	qPCR for U6 (housekeeping control)	Metabion	*(80)*
Random hexamers	-	Reverse transcription of U6	Metabion	*(17)*
TERRA-ASO (LNA Gapmer targetinng TERRA)	TAACCCTAACCCTAAC	TERRA depletion	Exiqon	*(43)*
LNA Gapmer SCRAMBLED (ASO SCR)	AACACGTCTATACGC	Control for TERRA depletion	Exiqon	*(43)*

Table S3. List of oligonucleotides used in this study.

Legends to Supplementary Movies

Supplementary Movies 1 and 2: TERRA-telomere colocalization in TERRA-MS2 cells, related to figure 6. Z-stack images of TRF1-mCherry (telomeres, red) and MCP-sfGFP (TERRA, green) were acquired every 60 seconds for 10 minutes. Image planes with visible TERRA foci from the timelapse movies. (Scale bar = 5 µm).

Supplementary Movies 3 and 4: hTR-telomere colocalization in hTR$^{5'MS2}$ cells transfected with ASO SCR, related to figure 8. Images of MCP-sfGFP hTR$^{5'MS2}$ (telomerase, green) and TRF1-mCherry (telomeres, red) were acquired every 5 seconds for 5 minutes. (Scale bar = 5 µm).

Supplementary Movies 5 and 6: hTR-telomere colocalization in hTR$^{5'MS2}$ cells transfected with TERRA-ASO, related to figure 8. Images of MCP-sfGFP hTR$^{5'MS2}$ (telomerase, green) and TRF1-mCherry (telomeres, red) were acquired every 5 seconds for 5 minutes. (Scale bar = 5 µm).

REFERENCES AND NOTES

1. C. M. Azzalin, P. Reichenbach, L. Khoriauli, E. Giulotto, J. Lingner, Telomeric repeat containing RNA and RNA surveillance factors at mammalian chromosome ends. *Science* **318**, 798–801 (2007).

2. S. Schoeftner, M. A. Blasco, Developmentally regulated transcription of mammalian telomeres by DNA-dependent RNA polymerase II. *Nat. Cell Biol.* **10**, 228–236 (2008).

3. B. Luke, A. Panza, S. Redon, N. Iglesias, Z. Li, J. Lingner, The Rat1p 5′ to 3′ exonuclease degrades telomeric repeat-containing RNA and promotes telomere elongation in Saccharomyces cerevisiae. *Mol. Cell* **32**, 465–477 (2008).

4. C. Manzato, L. Larini, C. O. Pegorar, M. R. Dello Stritto, K. Jurikova, V. Jantsch, E. Cusanelli, TERRA expression is regulated by the telomere-binding proteins POT-1 and POT-2 in Caenorhabditis elegans. *Nucleic Acids Res.* **51**, 10681–10699 (2023).

5. A. Diman, A. Decottignies, Genomic origin and nuclear localization of TERRA telomeric repeat-containing RNA: From darkness to dawn. *FEBS J.* **285**, 1389–1398 (2018).

6. A. Barral, J. Dejardin, Telomeric chromatin and TERRA. *J. Mol. Biol.* **432**, 4244–4256 (2020).

7. S. G. Nergadze, B. O. Farnung, H. Wischnewski, L. Khoriauli, V. Vitelli, R. Chawla, E. Giulotto, C. M. Azzalin, CpG-island promoters drive transcription of human telomeres. *RNA* **15**, 2186–2194 (2009).

8. A. Porro, S. Feuerhahn, J. Delafontaine, H. Riethman, J. Rougemont, J. Lingner, Functional characterization of the TERRA transcriptome at damaged telomeres. *Nat. Commun.* **5**, 5379 (2014).

9. J. Rivosecchi, K. Jurikova, E. Cusanelli, Telomere-specific regulation of TERRA and its impact on telomere stability. *Semin. Cell Dev. Biol.* **157**, 3–23 (2024).

10. I. Lopez de Silanes, M. Stagno d'Alcontres, M. A. Blasco, TERRA transcripts are bound by a complex array of RNA-binding proteins. *Nat. Commun.* **1**, 33 (2010).

11. Z. Deng, J. Norseen, A. Wiedmer, H. Riethman, P. M. Lieberman, TERRA RNA binding to TRF2 facilitates heterochromatin formation and ORC recruitment at telomeres. *Mol. Cell* **35**, 403–413 (2009).

12. E. Cusanelli, P. Chartrand, Telomeric noncoding RNA: Telomeric repeat-containing RNA in telomere biology. *Wiley Interdiscip. Rev. RNA* **5**, 407–419 (2014).

13. E. Cusanelli, C. A. Romero, P. Chartrand, Telomeric noncoding RNA TERRA is induced by telomere shortening to nucleate telomerase molecules at short telomeres. *Mol. Cell* **51**, 780–791 (2013).

14. M. Feretzaki, M. Pospisilova, R. Valador Fernandes, T. Lunardi, L. Krejci, J. Lingner, RAD51-dependent recruitment of TERRA lncRNA to telomeres through R-loops. *Nature* **587**, 303–308 (2020).

15. C. M. Azzalin, J. Lingner, Telomeres: The silence is broken. *Cell Cycle* **7**, 1161–1165 (2008).

16. A. Porro, S. Feuerhahn, P. Reichenbach, J. Lingner, Molecular dissection of telomeric repeat-containing RNA biogenesis unveils the presence of distinct and multiple regulatory pathways. *Mol. Cell. Biol.* **30**, 4808–4817 (2010).

17. V. Savoca, J. Rivosecchi, A. Gaiatto, A. Rossi, R. Mosca, I. Gialdini, L. Zubovic, T. Tebaldi, P. Macchi, E. Cusanelli, TERRA stability is regulated by RALY and polyadenylation in a telomere-specific manner. *Cell Rep.* **42**, 112406 (2023).

18. L. Chen, C. Zhang, W. Ma, J. Huang, Y. Zhao, H. Liu, METTL3-mediated m6A modification stabilizes TERRA and maintains telomere stability. *Nucleic Acids Res.* **50**, 11619–11634 (2022).

19. R. Vaid, K. Thombare, A. Mendez, R. Burgos-Panadero, A. Djos, D. Jachimowicz, K. I. Lundberg, C. Bartenhagen, N. Kumar, C. Tummler, C. Sihlbom, S. Fransson, J. I. Johnsen, P. Kogner, T. Martinsson, M. Fischer, T. Mondal, METTL3 drives telomere targeting of TERRA lncRNA through m6A-dependent R-loop formation: A therapeutic target for ALT-positive neuroblastoma. *Nucleic Acids Res.* **52**, 2648–2671 (2024).

20. N. Arnoult, A. Van Beneden, A. Decottignies, Telomere length regulates TERRA levels through increased trimethylation of telomeric H3K9 and HP1α. *Nat. Struct. Mol. Biol.* **19**, 948–956 (2012).

21. R. L. Flynn, R. C. Centore, R. J. O'Sullivan, R. Rai, A. Tse, Z. Songyang, S. Chang, J. Karlseder, L. Zou, TERRA and hnRNPA1 orchestrate an RPA-to-POT1 switch on telomeric single-stranded DNA. *Nature* **471**, 532–536 (2011).

22. K. Beishline, O. Vladimirova, S. Tutton, Z. Wang, Z. Deng, P. M. Lieberman, CTCF driven TERRA transcription facilitates completion of telomere DNA replication. *Nat. Commun.* **8**, 2114 (2017).

23. A. Porro, S. Feuerhahn, J. Lingner, TERRA-reinforced association of LSD1 with MRE11 promotes processing of uncapped telomeres. *Cell Rep.* **6**, 765–776 (2014).

24. N. Hug, J. Lingner, Telomere length homeostasis. *Chromosoma* **115**, 413–425 (2006).

25. F. d'Adda di Fagagna, P. M. Reaper, L. Clay-Farrace, H. Fiegler, P. Carr, T. Von Zglinicki, G. Saretzki, N. P. Carter, S. P. Jackson, A DNA damage checkpoint response in telomere-initiated senescence. *Nature* **426**, 194–198 (2003).

26. C. W. Greider, E. H. Blackburn, Identification of a specific telomere terminal transferase activity in Tetrahymena extracts. *Cell* **43**, 405–413 (1985).

27. J. C. Schmidt, T. R. Cech, Human telomerase: Biogenesis, trafficking, recruitment, and activation. *Genes Dev.* **29**, 1095–1105 (2015).

28. N. W. Kim, M. A. Piatyszek, K. R. Prowse, C. B. Harley, M. D. West, P. L. Ho, G. M. Coviello, W. E. Wright, S. L. Weinrich, J. W. Shay, Specific association of human telomerase activity with immortal cells and cancer. *Science* **266**, 2011–2015 (1994).

29. J. Nandakumar, C. F. Bell, I. Weidenfeld, A. J. Zaug, L. A. Leinwand, T. R. Cech, The TEL patch of telomere protein TPP1 mediates telomerase recruitment and processivity. *Nature* **492**, 285–289 (2012).

30. F. L. Zhong, L. F. Batista, A. Freund, M. F. Pech, A. S. Venteicher, S. E. Artandi, TPP1 OB-fold domain controls telomere maintenance by recruiting telomerase to chromosome ends. *Cell* **150**, 481–494 (2012).

31. J. C. Schmidt, A. J. Zaug, R. Kufer, T. R. Cech, Dynamics of human telomerase recruitment depend on template-telomere base pairing. *Mol. Biol. Cell* **29**, 869–880 (2018).

32. T. de Lange, Shelterin-mediated telomere protection. *Annu. Rev. Genet.* **52**, 223–247 (2018).

33. P. Baumann, T. R. Cech, Pot1, the putative telomere end-binding protein in fission yeast and humans. *Science* **292**, 1171–1175 (2001).

34. D. Loayza, T. De Lange, POT1 as a terminal transducer of TRF1 telomere length control. *Nature* **423**, 1013–1018 (2003).

35. C. M. Latrick, T. R. Cech, POT1-TPP1 enhances telomerase processivity by slowing primer dissociation and aiding translocation. *EMBO J.* **29**, 924–933 (2010).

36. F. Wang, E. R. Podell, A. J. Zaug, Y. Yang, P. Baciu, T. R. Cech, M. Lei, The POT1-TPP1 telomere complex is a telomerase processivity factor. *Nature* **445**, 506–510 (2007).

37. H. Laprade, E. Querido, M. J. Smith, D. Guerit, H. Crimmins, D. Conomos, E. Pourret, P. Chartrand, A. Sfeir, Single-molecule imaging of telomerase RNA reveals a recruitment-retention model for telomere elongation. *Mol. Cell* **79**, 115–126.e6 (2020).

38. Y. Zhao, A. J. Sfeir, Y. Zou, C. M. Buseman, T. T. Chow, J. W. Shay, W. E. Wright, Telomere extension occurs at most chromosome ends and is uncoupled from fill-in in human cancer cells. *Cell* **138**, 463–475 (2009).

39. J. C. Schmidt, A. J. Zaug, T. R. Cech, Live cell imaging reveals the dynamics of telomerase recruitment to telomeres. *Cell* **166**, 1188–1197.e9 (2016).

40. M. Moravec, H. Wischnewski, A. Bah, Y. Hu, N. Liu, L. Lafranchi, M. C. King, C. M. Azzalin, TERRA promotes telomerase-mediated telomere elongation in Schizosaccharomyces pombe. *EMBO Rep.* **17**, 999–1012 (2016).

41. S. Redon, P. Reichenbach, J. Lingner, The non-coding RNA TERRA is a natural ligand and direct inhibitor of human telomerase. *Nucleic Acids Res.* **38**, 5797–5806 (2010).

42. S. Redon, I. Zemp, J. Lingner, A three-state model for the regulation of telomerase by TERRA and hnRNPA1. *Nucleic Acids Res.* **41**, 9117–9128 (2013).

43. H. P. Chu, C. Cifuentes-Rojas, B. Kesner, E. Aeby, H. G. Lee, C. Wei, H. J. Oh, M. Boukhali, W. Haas, J. T. Lee, TERRA RNA antagonizes ATRX and protects telomeres. *Cell* **170**, 86–101.e16 (2017).

44. B. O. Farnung, C. M. Brun, R. Arora, L. E. Lorenzi, C. M. Azzalin, Telomerase efficiently elongates highly transcribing telomeres in human cancer cells. *PLOS ONE* **7**, e35714 (2012).

45. S. Sagie, E. Ellran, H. Katzir, R. Shaked, S. Yehezkel, I. Laevsky, A. Ghanayim, D. Geiger, M. Tzukerman, S. Selig, Induced pluripotent stem cells as a model for telomeric abnormalities in ICF type I syndrome. *Hum. Mol. Genet.* **23**, 3629–3640 (2014).

46. S. Yehezkel, A. Rebibo-Sabbah, Y. Segev, M. Tzukerman, R. Shaked, I. Huber, L. Gepstein, K. Skorecki, S. Selig, Reprogramming of telomeric regions during the generation of human induced pluripotent stem cells and subsequent differentiation into fibroblast-like derivatives. *Epigenetics* **6**, 63–75 (2011).

47. B. Ma, P. Martinez, R. Sanchez-Vazquez, M. A. Blasco, Telomere dynamics in human pluripotent stem cells. *Cell Cycle* **22**, 2505–2521 (2023).

48. R. M. Marion, K. Strati, H. Li, A. Tejera, S. Schoeftner, S. Ortega, M. Serrano, M. A. Blasco, Telomeres acquire embryonic stem cell characteristics in induced pluripotent stem cells. *Cell Stem Cell* **4**, 141–154 (2009).

49. J. Rivosecchi, E. Cusanelli, TERRA beyond cancer: The biology of telomeric repeat-containing RNAs in somatic and germ cells. *Front. Aging* **4**, 1224225 (2023).

50. R. L. Tomlinson, T. D. Ziegler, T. Supakorndej, R. M. Terns, M. P. Terns, Cell cycle-regulated trafficking of human telomerase to telomeres. *Mol. Biol. Cell* **17**, 955–965 (2006).

51. B. E. Jady, P. Richard, E. Bertrand, T. Kiss, Cell cycle-dependent recruitment of telomerase RNA and Cajal bodies to human telomeres. *Mol. Biol. Cell* **17**, 944–954 (2006).

52. E. Querido, A. Sfeir, P. Chartrand, Imaging of telomerase RNA by single-molecule inexpensive FISH combined with immunofluorescence. *STAR Protoc.* **1**, 100104 (2020).

53. N. Tsanov, A. Samacoits, R. Chouaib, A. M. Traboulsi, T. Gostan, C. Weber, C. Zimmer, K. Zibara, T. Walter, M. Peter, E. Bertrand, F. Mueller, smiFISH and FISH-quant—A flexible single RNA detection approach with super-resolution capability. *Nucleic Acids Res.* **44**, e165 (2016).

54. B. Li, S. Oestreich, T. de Lange, Identification of human Rap1. *Cell* **101**, 471–483 (2000).

55. B. Silva, R. Pentz, A. M. Figueira, R. Arora, Y. W. Lee, C. Hodson, H. Wischnewski, A. J. Deans, C. M. Azzalin, FANCM limits ALT activity by restricting telomeric replication stress induced by deregulated BLM and R-loops. *Nat. Commun.* **10**, 2253 (2019).

56. K. Damm, U. Hemmann, P. Garin-Chesa, N. Hauel, I. Kauffmann, H. Priepke, C. Niestroj, C. Daiber, B. Enenkel, B. Guilliard, I. Lauritsch, E. Muller, E. Pascolo, G. Sauter, M. Pantic, U. M. Martens, C. Wenz, J. Lingner, N. Kraut, W. J. Rettig, A. Schnapp, A highly selective telomerase inhibitor limiting human cancer cell proliferation. *EMBO J.* **20**, 6958–6968 (2001).

57. M. Graf, D. Bonetti, A. Lockhart, K. Serhal, V. Kellner, A. Maicher, P. Jolivet, M. T. Teixeira, B. Luke, Telomere length determines TERRA and R-loop regulation through the cell cycle. *Cell* **170**, 72–85.e14 (2017).

58. P. M. Lansdorp, N. P. Verwoerd, F. M. van de Rijke, V. Dragowska, M. T. Little, R. W. Dirks, A. K. Raap, H. J. Tanke, Heterogeneity in telomere length of human chromosomes. *Hum. Mol. Genet.* **5**, 685–691 (1996).

59. S. Henderson, R. Allsopp, D. Spector, S. S. Wang, C. Harley, In situ analysis of changes in telomere size during replicative aging and cell transformation. *J. Cell Biol.* **134**, 1–12 (1996).

60. A. Vancevska, K. M. Douglass, V. Pfeiffer, S. Manley, J. Lingner, The telomeric DNA damage response occurs in the absence of chromatin decompaction. *Genes Dev.* **31**, 567–577 (2017).

61. L. Avogaro, C. Oss Pegorar, N. Bettin, E. Cusanelli, Generation of cancer cell clones to visualize telomeric repeat-containing RNA TERRA expressed from a single telomere in living cells. *J. Vis. Exp.* **143**, e58790 (2019).

62. L. Avogaro, E. Querido, M. Dalachi, M. F. Jantsch, P. Chartrand, E. Cusanelli, Live-cell imaging reveals the dynamics and function of single-telomere TERRA molecules in cancer cells. *RNA Biol.* **15**, 787–796 (2018).

63. S. Yehezkel, Y. Segev, E. Viegas-Pequignot, K. Skorecki, S. Selig, Hypomethylation of subtelomeric regions in ICF syndrome is associated with abnormally short telomeres and enhanced transcription from telomeric regions. *Hum. Mol. Genet.* **17**, 2776–2789 (2008).

64. B. Chen, L. A. Gilbert, B. A. Cimini, J. Schnitzbauer, W. Zhang, G. W. Li, J. Park, E. H. Blackburn, J. S. Weissman, L. S. Qi, B. Huang, Dynamic imaging of genomic loci in living human cells by an optimized CRISPR/Cas system. *Cell* **155**, 1479–1491 (2013).

65. K. Okada, H. Sugihara, M. Bamba, T. Bamba, T. Hattori, Sequential numerical changes of chromosomes 7 and 18 in diffuse-type stomach cancer cell lines: Combined comparative genomic hybridization, fluorescence in situ hybridization, and ploidy analyses. *Cancer Genet. Cytogenet.* **118**, 99–107 (2000).

66. J. Y. Tinevez, N. Perry, J. Schindelin, G. M. Hoopes, G. D. Reynolds, E. Laplantine, S. Y. Bednarek, S. L. Shorte, K. W. Eliceiri, TrackMate: An open and extensible platform for single-particle tracking. *Methods* **115**, 80–90 (2017).

67. N. Kaminski, A. R. Wondisford, Y. Kwon, M. L. Lynskey, R. Bhargava, J. Barroso-Gonzalez, L. Garcia-Exposito, B. He, M. Xu, D. Mellacheruvu, S. C. Watkins, M. Modesti, K. M. Miller, A. I. Nesvizhskii, H. Zhang, P. Sung, R. J. O'Sullivan, RAD51AP1 regulates ALT-HDR through chromatin-directed homeostasis of TERRA. *Mol. Cell* **82**, 4001–4017.e7 (2022).

68. T. Yadav, J. M. Zhang, J. Ouyang, W. Leung, A. Simoneau, L. Zou, TERRA and RAD51AP1 promote alternative lengthening of telomeres through an R- to D-loop switch. *Mol. Cell* **82**, 3985–4000.e4 (2022).

69. M. Lalonde, P. Chartrand, TERRA, a multifaceted regulator of telomerase activity at telomeres. *J. Mol. Biol.* **432**, 4232–4243 (2020).

70. S. Marcand, E. Gilson, D. Shore, A protein-counting mechanism for telomere length regulation in yeast. *Science* **275**, 986–990 (1997).

71. B. Britt-Compton, R. Capper, J. Rowson, D. M. Baird, Short telomeres are preferentially elongated by telomerase in human cells. *FEBS Lett.* **583**, 3076–3080 (2009).

72. G. Chen, X. Deng, Cell synchronization by double thymidine block. *Bio Protoc.* **8**, e2994 (2018).

73. R. Zufferey, D. Nagy, R. J. Mandel, L. Naldini, D. Trono, Multiply attenuated lentiviral vector achieves efficient gene delivery in vivo. *Nat. Biotechnol.* **15**, 871–875 (1997).

74. J. Ollion, J. Cochennec, F. Loll, C. Escude, T. Boudier, TANGO: A generic tool for high-throughput 3D image analysis for studying nuclear organization. *Bioinformatics* **29**, 1840–1841 (2013).

75. J. F. Gilles, M. Dos Santos, T. Boudier, S. Bolte, N. Heck, DiAna, an ImageJ tool for object-based 3D co-localization and distance analysis. *Methods* **115**, 55–64 (2017).

76. F. Rossiello, M. Fumagalli, F. D'Adda di Fagagna, ImmunoFISH for adherent cultured mammalian cells. *Bio Protoc.* **3**, e999 (2013).

77. H. P. Chu, J. E. Froberg, B. Kesner, H. J. Oh, F. Ji, R. Sadreyev, S. F. Pinter, J. T. Lee, PAR-TERRA directs homologous sex chromosome pairing. *Nat. Struct. Mol. Biol.* **24**, 620–631 (2017).

78. C. K. Tseng, H. F. Wang, A. M. Burns, M. R. Schroeder, M. Gaspari, P. Baumann, Human telomerase RNA processing and quality control. *Cell Rep.* **13**, 2232–2243 (2015).

79. L. Chen, C. M. Roake, A. Galati, F. Bavasso, E. Micheli, I. Saggio, S. Schoeftner, S. Cacchione, M. Gatti, S. E. Artandi, G. D. Raffa, Loss of human TGS1 hypermethylase promotes increased telomerase RNA and telomere elongation. *Cell Rep.* **30**, 1358–1372.e5 (2020).

80. B. Zhang, L. Gunawardane, F. Niazi, F. Jahanbani, X. Chen, S. Valadkhan, A novel RNA motif mediates the strict nuclear localization of a long noncoding RNA. *Mol. Cell. Biol.* **34**, 2318–2329 (2014).

81. Z. Deng, Z. Wang, N. Stong, R. Plasschaert, A. Moczan, H. S. Chen, S. Hu, P. Wikramasinghe, R. V. Davuluri, M. S. Bartolomei, H. Riethman, P. M. Lieberman, A role for CTCF and cohesin in subtelomere chromatin organization, TERRA transcription, and telomere end protection. *EMBO J.* **31**, 4165–4178 (2012).

82. M. Feretzaki, J. Lingner, A practical qPCR approach to detect TERRA, the elusive telomeric repeat-containing RNA. *Methods* **114**, 39–45 (2017).
